# Geospatial modelling of farmer–herder interactions maps cultural geography of Bronze and Iron Age Tibet, 3600–2200 BP

**DOI:** 10.1038/s41598-023-50556-9

**Published:** 2024-02-02

**Authors:** Xinzhou Chen, Hongliang Lü, Xinyi Liu, Michael D. Frachetti

**Affiliations:** 1 Center for Archaeological Sciences, Sichuan University, Chengdu, Sichuan China; 2 SAIE Laboratory, Department of Anthropology, Washington University in St. Louis, St. Louis, MO USA; 3 School of Cultural Heritage, Northwest University, Xi’an, China

**Keywords:** Socioeconomic scenarios, Behavioural ecology

## Abstract

Tibetan cultures reflect deeply rooted, regional interactions and diverse subsistence practices across varied high-altitude environments of the Tibetan Plateau. Yet, it remains unclear how these cultural relationships and social interactions took shape through time and how they were influenced by ecologically oriented behavioral strategies (e.g. mobility) emerging in prehistory. Recent applications of network analysis provide novel tools to quantitatively measure shared forms of material culture, but there have been fewer attempts to couple social network analysis with fine-grained geospatial modelling of prehistoric human mobility in Tibet. In this study, we developed an integrated high-resolution geospatial model and network analysis that simulates and correlates subsistence-based mobility and ceramic-based cultural material connectivity across the Tibetan Plateau. Our analysis suggests that (1) ecologically driven patterns of subsistence-based mobility correspond geographically with Bronze and Iron Ages settlement patterns across the Tibetan Plateau; (2) diverse material interaction networks among communities within western and central Tibet and trans-Himalayan connectivity across the broader Inner Asian Mountain Corridor can be linked to modeled differences in regional networks of subsistence mobility. This research provides ecological and archaeological insights into how subsistence-oriented mobility and interaction may have shaped documented patterns of social and material connectivity among regional Bronze and Iron Age communities of the Tibetan Plateau, prompting a reconsideration of Tibet's long-term cultural geography.

## Introduction

Situated at the heart of the Eurasian continent, the Tibetan Plateau hosts the world’s largest high-altitude ecosystem and is home to a diversity of cultures in the past and present. With over 70% of the landmass covered by grassland, farming and herding play major roles in the economy of the Tibetan Plateau today^1^. Through more than 70 years of archaeological research on the plateau, scholars have exposed regional facets of its archaeological material cultures, indicating different degrees of cultural connectivity among populations within and beyond the Tibetan Plateau during the Bronze and Iron Ages (3600–2200 BP)^2,3^. Recent archaeological research also sheds much-needed light on the prehistoric economy of the plateau in a context of increasingly globalized foodways, after ca. 3600 BP, with increasing reliance on cereal crops such as barley and millet and the use of ruminant animals, such as yak, taurine cattle, horse, goat, and sheep^3–5^. The emergence of diverse foodways and the flexibility of strategies among farmers, agropastoralists and pastoral nomads enabled the inhabitation of marginal environments across the Tibetan Plateau during this time^6,7^. Little is known, however, about the underlying mechanisms that shaped the settlement pattern and the nature of prehistoric social interactions in the Bronze and Iron Ages on the Plateau.

In this study, we aim to investigate how economically motivated mobility corresponding to mixed farming and herding strategies contributed to trans-regional material exchange (e.g., ceramics) in prehistory. We seek to understand how the geospatial patterning of interactions—modelled as a mobility network between agrarian zones and highland pastures—correlates with the evident geography of human settlement and the material connections among Tibetan communities throughout the Bronze and Iron Ages^8^. Comparing the geographies of subsistence based-mobility with a comprehensive qualitative (typological) assessment of Bronze and Iron Age ceramic artifacts, this research aims to provide an intersectional interpretative framework that explores cultural diversification and regional connectivity in the Bronze and Iron Ages across the Tibetan Plateau.

The varying ecozones of the Tibetan Plateau, including mountains, steppes, and river valleys, played a role in determining site locations and related social interactions throughout later prehistory. Majiayao cultural groups were mainly situated along the foothills of the northeastern margin of the Tibetan Plateau during the Neolithic period (approximately ca. 6000–4200 BP). These were millet farmers who utilized broad subsistence strategies including pig rearing and foraging/hunting, with settlements mostly situated along the Qinghai Mountains and the Hexi Corridor^6^. The cultural/dietary character of groups associated with Majiayao, Zongri and other regional groups, subsequently spread towards the eastern Tibetan Plateau, penetrating Western Sichuan and Northwestern Yunnan^9,10^. Ecologically suitable niches for mixed economies provided optimal pathways for the spread of human settlements, material traditions, and technologies in the eastern, southern and central Tibetan Plateau during this time. On the northeastern plateau, the post Majiayao cultures, including Qijia, mark a florescence of new and cultural traditions with varying forms of pastoralism in northeastern Tibet^11^. After the fourth millennium BP, agropastoral settlements further expanded into high-altitude and ecologically challenging environments across the Tibetan Plateau^3^.

In Central and Western Tibet, the material culture after ca. 3600 BP also underwent significant changes, featuring new ceramic traditions, metal artifacts and new burial traditions^12,13^. Previous research commonly attributed the changes in material culture and subsistence economies to increasing human mobility, trans-regional material exchange and environmental changes in the Bronze and Iron Age Eurasia^3,6^. Current archaeological research points to the fact that the ancient people on the Tibetan Plateau intensively participated in different geographical and social networks^14^, allowing us to examine the relationships among the human settlement pattern, subsistence strategies and sociocultural interactions.

To assess the geography of interactions between communities (and sub-communities) of farmers and herders, our model uses modelled vegetative productivity and land use as a prime factor to map potential mobility networks. Different from previous research which uses slope-derived mobility models based on the least-cost path methods^14,15^, we assume that ancient highland herders moved regularly towards lowland arable niches using rich-vegetal niches and high-quality pastures in the mountains and steppes, instead of moving through valley bottoms, per se. This resonates with the general movement pattern of contemporary agropastoral communities that are documented by ethnographic work and attested in archaeological studies^16,17^.

We model mobility using the flow accumulation method to simulate human movements as integrated flows across a cost surface^18^, which has been successfully used to analyze highland pastoralist mobility in Central Asia^17^. By aggregating all accumulated flows between productive highland patches and arable lands, we generated a Subsistence Interaction Mobility Model (SIMM) where the entire Tibetan Plateau is coded with different values indicating the hypothesized ‘traffic’ volumes of movement among agropastoral communities.

Of note, current archaeological evidence demonstrates that ancient human communities practiced mixed farming and herding in most archaeological sites discovered to date. By simulating movement across high vegetal patches toward arable lands, the SIMM characterizes interactions within two interrelated subsistence modalities distributed across different landscapes, rather than a simplified interface between idealized human communities with distinctive economies. The simulated routes of the SIMM were then compared geo-statistically with 1434 Bronze and Iron Age sites of Tibet, geolocated from published archaeological research.

**Figure 1:**
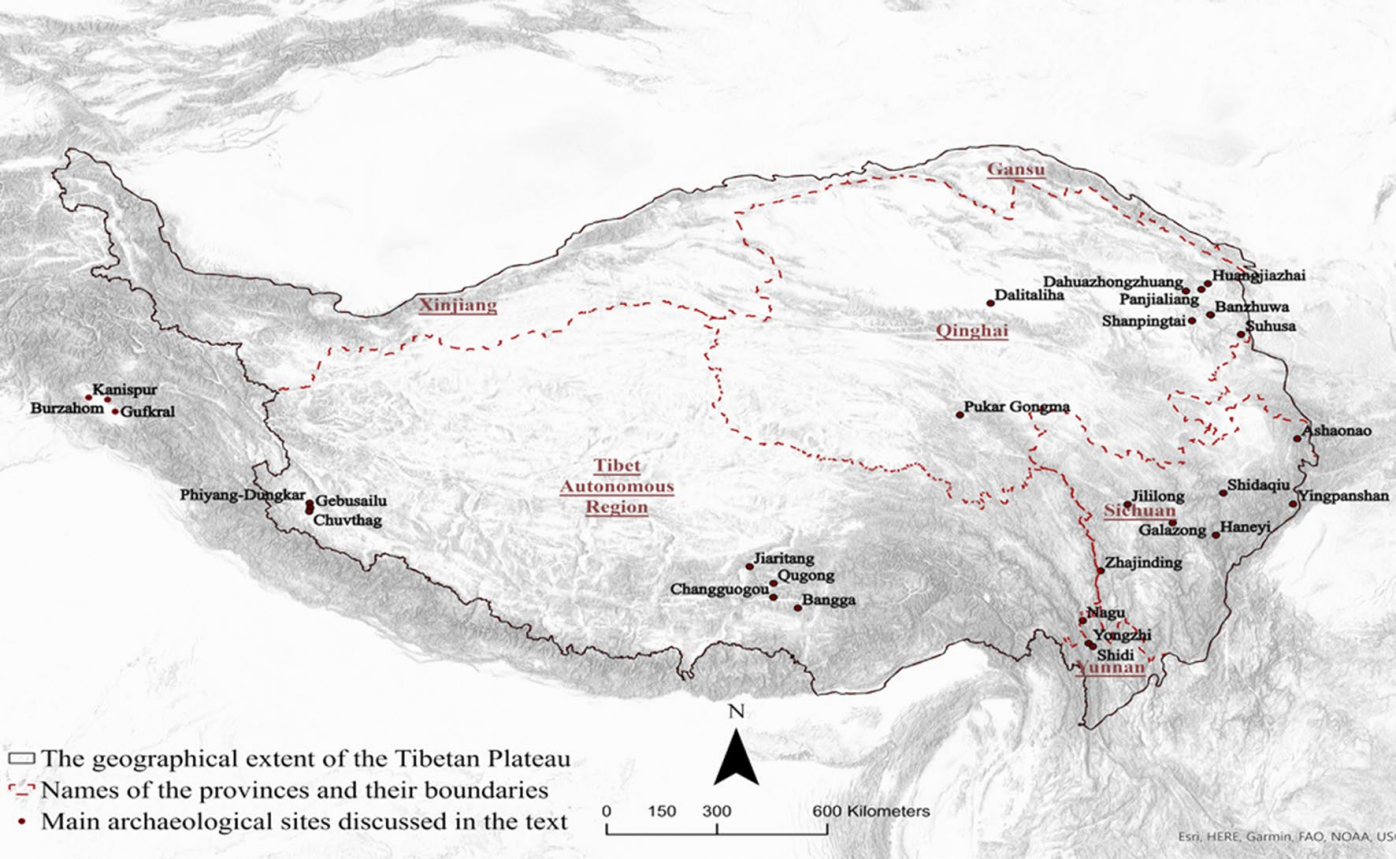
The geography of Tibet and the main sites discussed in the text.

To evaluate the degree to which the SIMM pathways correlate with archaeological evidence for social interactions, we also generated a social network based on the similarity of the archaeological ceramics from 26 sites between ca. 3600–2200 BP (Fig. 1). The ceramic network is constructed based on the presence and absence of 51 morphological attributes, which serves as an archaeological proxy for the intensity and geographical structure of social interactions. We quantitatively compared the SIMM with the locations of archaeological sites on the Tibetan Plateau and the ceramic network, exploring the relationships among subsistence-based mobility, settlement patterns and social interactions. To investigate the potential mechanism that may have driven the patterns of the simulated geographical and cultural connectivity and compensating for the limitations of quantitative analysis in characterizing the nuances of inter-regional interactions, we further discuss the similarities and differences between networks based on typological analysis of the cultural artifacts in Tibetan archaeology.

## Results

### Modelling farmer–herder interactions: subsistence interaction mobility model (SIMM)

Ethnographic, historical, and archaeological research of Tibet and its surrounding regions provides a rich account of the farmer–herder interactions that enables us to broadly simulate their mobility patterns in a geospatial model. Ethnographic research documents that herders frequently interact with farmers for trade and information exchange (and vice versa). For example, Goldstein and Beall documented the importance of trade with farmers for the contemporary Phala nomads in Changtang (Northern Tibet)^16^. The Phala nomads usually travel long distances for 20–30 days to lowland agropastoral villages and barter the byproducts of their livestock for grain. In Eastern Tibet, ethnographical records also documented the seasonal movement circle of Dora Karmo nomads in detail, the pathways of which are dictated by the high grazing need of yaks^19^. For the lowland agropastoralists in Tibet, herders travel between mountain pastures and lowland agricultural homesteads on a daily basis, most of which are short-distance movements seeking pastures^20^. Archaeological research and historical research posited that long-term farmer–herder interactions were so frequent that may have driven the evolution of cultural identities and social cohesion in the frontiers of ancient China throughout prehistory and the historic period^21,22^.

Recent research on the geography of ancient Central Asian nomads suggests that optimal grassland ecology in the mountains underpins the herd-facilitated, seasonal pastoral movements in Central Asia, as herders travel through rough terrains frequently because of the existence of high-quality pasture in high-elevation eco-zones^17^. Previous research revealed two aspects of farmer–herder interactions in Asian highlands which can be incorporated into our geospatial model: (1) farming is concentrated mostly in arable lands, while herders tend to use a wider more varied pasture landscape, spanning mountains and steppes. Therefore, farmer–herder interaction mobility can be broadly spatialized as moving from areas of richer grassland vegetation toward arable lands (and vice versa); (2) farmers and herders interact frequently in the past and present and these mutual interactions are partially facilitated by the geography of pastures (for feeding pack animals and herds). Thus, the pathways of farmer–herder interactions can be broadly modelled as routes that maximize high vegetal patches instead of routes across flat terrain with low slope values.

**Figure 2:**
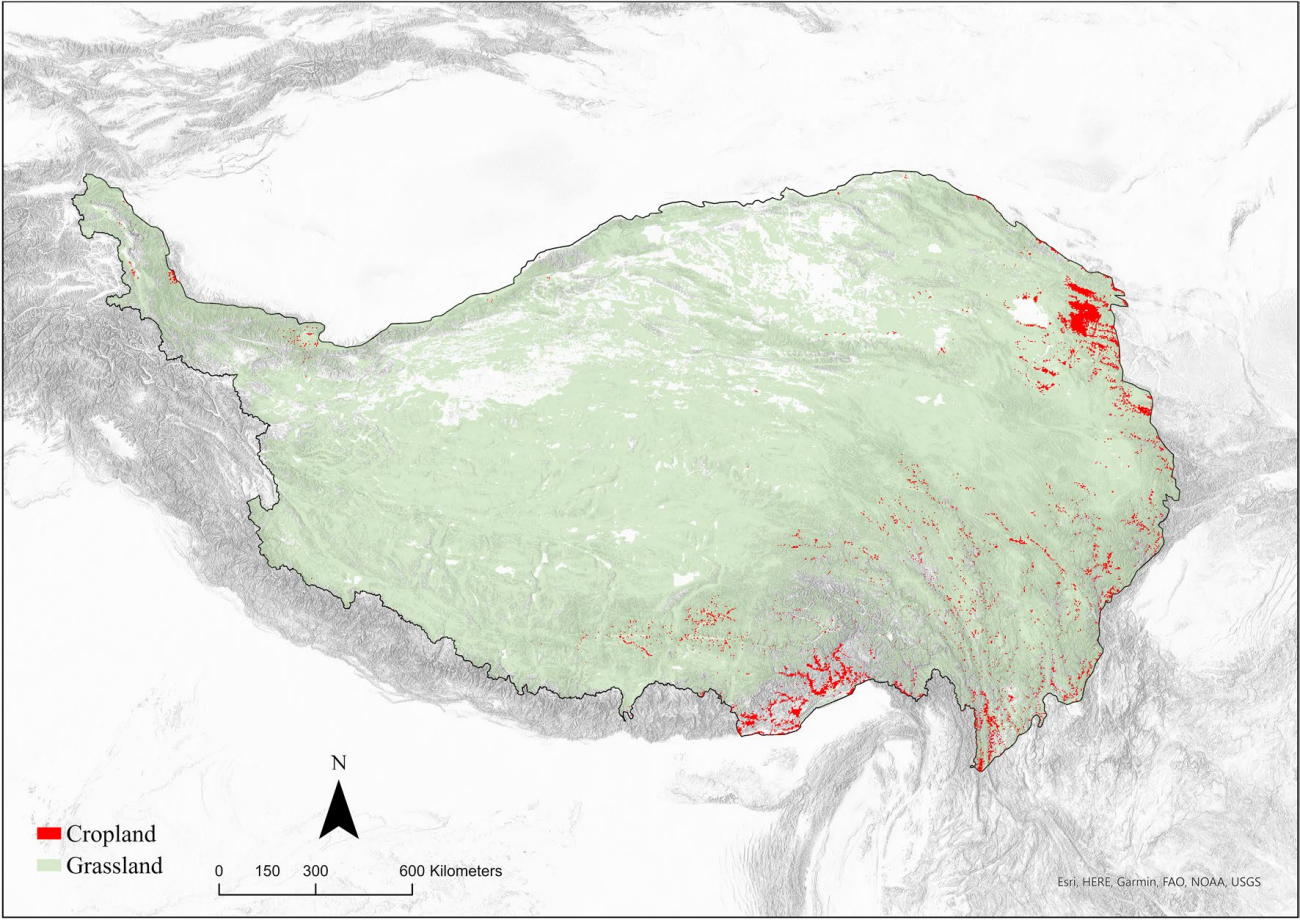
The distribution of modern grassland and cropland on the Tibetan Plateau (Source: Food and Agriculture Organization^23^).

In this context, since over 70 percent of the Tibetan Plateau is covered by different types of grasslands and bare soil according to remote sensing data and geological surveys^23^ (Fig. 2), we assume that the interactions between farmers and herders in Tibet may also have been facilitated by pasture quality after the introduction of herds and domesticated plants, and the geography of farmer–herder interactions can be broadly modelled as aggregate movements from non-croplands to arable lands via optimal vegetation and highland pastures^17^.

Each iteration of the Subsistence Interaction Mobility Model (SIMM) simulates optimal movement trajectories (flows) from all areas on the Tibetan Plateau to one cropland location, under the assumption that herders, for example, preferred trajectories that offered the best vegetation, a still common driver of herder mobility on the plateau based on ethnographic observations^16,19^. The model also iteratively changes the destinations to map toward all documented croplands (n = 6459) vectorized from modern satellite imagery. The resulting optimal “mobility highway” is generated after adding up all “pasture-to-croplands” pathways. For methodological details of the SIMM see Supplementary Methods Sects. 1.1 and 1.3.

**Figure 3:**
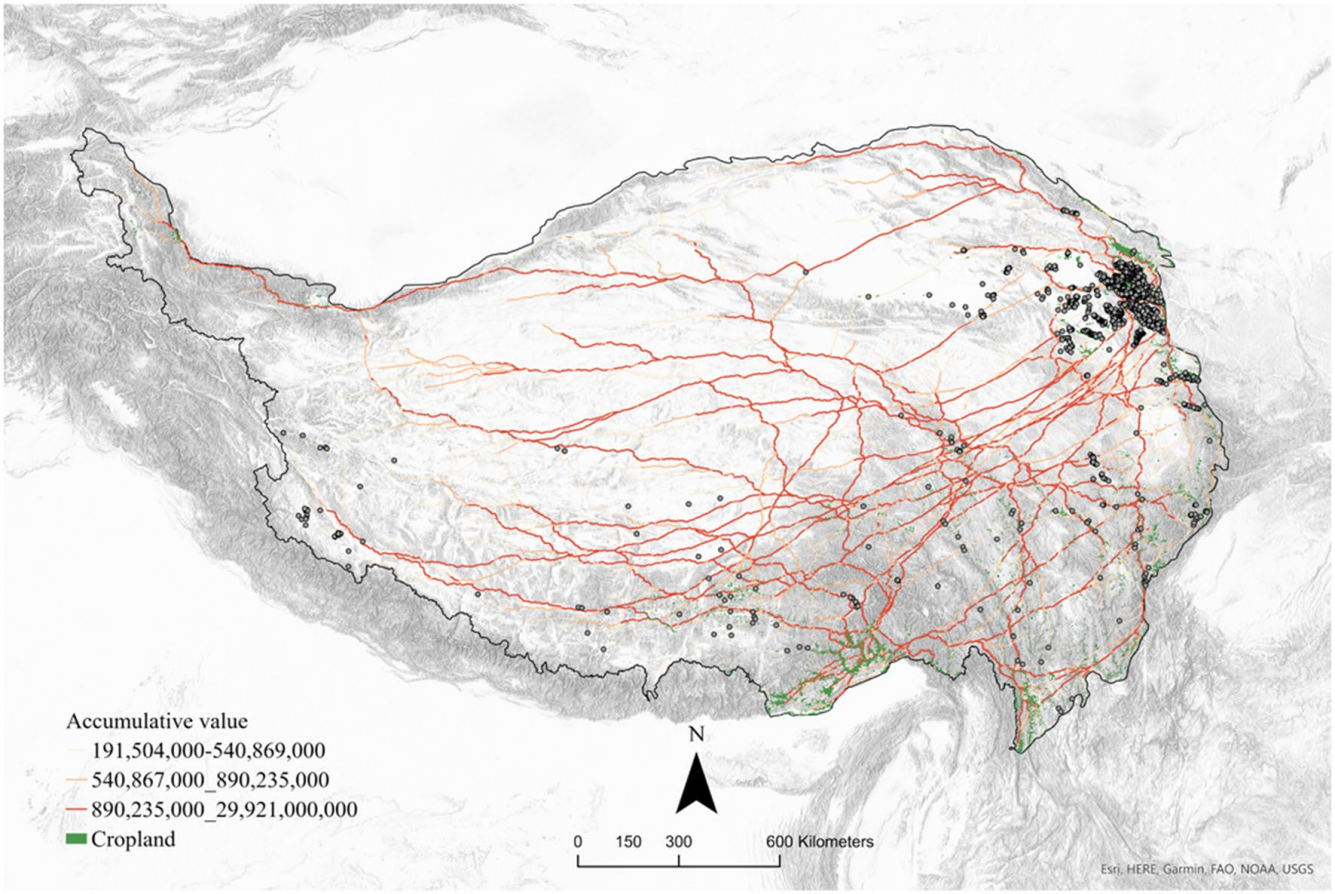
Simulated “mobility highways” of farmer–herder interactions overlaid with the geolocated archaeological sites dated between ca. 3600 and 2200 BP. The red line on the map indicates the pathways of the highest simulated “traffic volume”, The closer a site is to the “mobility highways”, the more efficient the flow of material, commodities, ideas, and information during its period of occupation. This map shows that the “mobility highways” are concentrated in the eastern part of the Tibetan Plateau.

The aggregated result of the model quantifies the relative potential of all subsistence interaction mobility with the flow accumulation values, where the most traversed areas (highest values) represent simulated “mobility highways” with extremely high traffic volume. We hypothesize that for any given area, the closer a site is to the “mobility highways”, the more efficient the flow of material, commodities, ideas and information during its period of occupation (Fig. 3).

**Figure 4:**
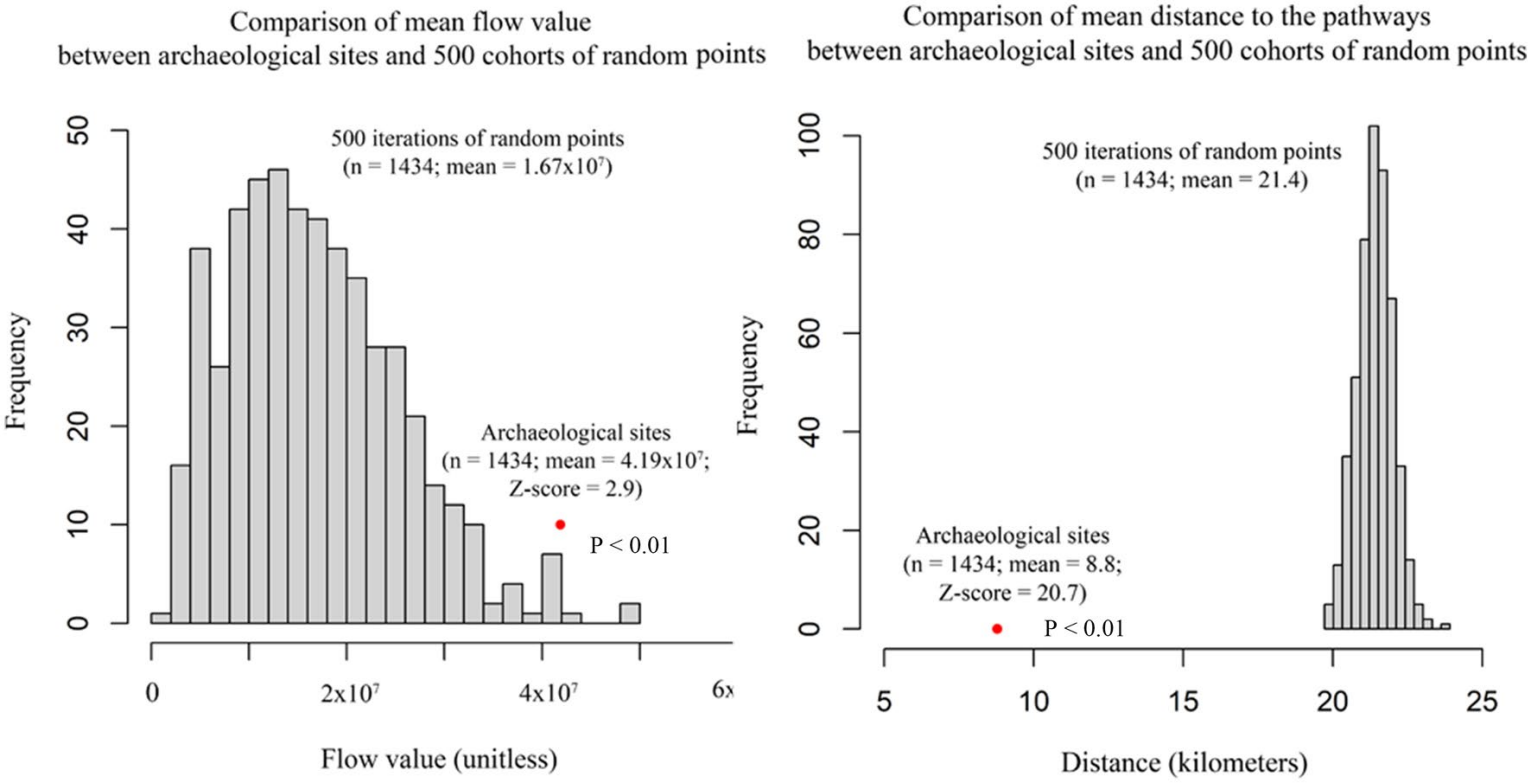
Comparison of mean flow value and distance to pathways between archaeological sites and 500 cohorts of random points. Z-scores measure how many standard deviations the mean values of archaeological points are below or above those of random points.

We compared the spatial and quantitative results of the SIMM using 1434 archaeological sites in the Bronze and Iron Age Tibet, geolocated from published research articles, archaeological reports and legacy archaeological surveys (Fig. 3). We also generated 500 cohorts of 1434 random points and compared their flow values and distances to the simulated “highways” with those of actual archaeological sites dating between ca. 3600 and 2200 BP (Fig. 4). The results suggest that the mean flow value of archaeological sites is significantly larger than the random points with a Z-score of 2.9 (for the calculation of Z-score see Methods section), illustrating the mean flow value of archaeological sites is larger than that of the random points by approximately three standard derivations. The mean distances to the simulated pathways of archaeological sites and random points are 8.8 km and 21.4 km, respectively, with a Z-score of 20.7. The evaluation of the SIMM and the statistical comparison of the Z-scores of mean flow values and distances to the pathways indicate that the archaeological sites are significantly closer (geographically) to the simulated pathways than random points (Fig. 4; for a fully annotated version of the statistical validation see Supplementary Methods Sect. 1.4 and Supplementary Fig. 4). We thus argue that ancient settlements were located to facilitate and benefit from intensified networks of mobility, information flow, and vegetative productivity across the Tibetan Plateau. To further test this argument, we also compared our results with a quantitative and qualitative analysis of Tibetan Bronze and Iron Age cultural artifacts. The comparison between the SIMM and cultural connectivity of archaeological artifacts requires a statistical transformation of the SIMM into a formal "subsistence interaction network" (or SIN, below), based on the geographical connectivity evident among real archaeological sites.

### The subsistence interaction network (SIN; derived from the SIMM)

The SIMM is a geospatial model based on environmental variables without using archaeological sites as input. To explore the geospatial connectivity of subsistence interaction among documented archaeological sites and how this simulated connectivity may have been correlated with the observed connectivity in archaeological materials, we converted the SIMM into a Subsistence Interaction Network (SIN) connecting 26 well-excavated archaeological sites dated between 3600 and 2200 BP using the “Least-Cost Path as Distance” function in ArcGIS Pro. The SIN is a matrix that calculates the degree to which pairwise sites were connected geographically when they use the “farmer–herder mobility highways” modelled in SIMM as much as possible. We use only 26 sites to compare their geographical and cultural connectivity because of a lack of well described ceramic assemblages and comprehensively published archaeological site reports in Tibet. Although those 26 sites are only a small subset in the complete dataset of mapped archaeological sites, we consider that this selection offers the most reliable data to use for baseline modelling of cultural connectivity, as they also provide both archaeologically and chronologically reliable material to model ceramic networks (below).

For the SIN, connectivity between archaeological sites is calculated using the shortest and highly weighted “flow distance” (derived from the SIMM) between any pair of sites. The ties among sites in the SIN measure the theoretical potential of interaction between sites if connectivity took shape along the simulated “highways”. For example, if two sites are in areas with high flow accumulation values (simulated traffic volume in the SIMM), they tend to have a high value of shortest “flow distance” in SIN, indicating a higher potential for mutual interactions. The network is visualized using an arbitrary cutoff value to remove weak ties between sites as the method permits^8^.

**Figure 5:**
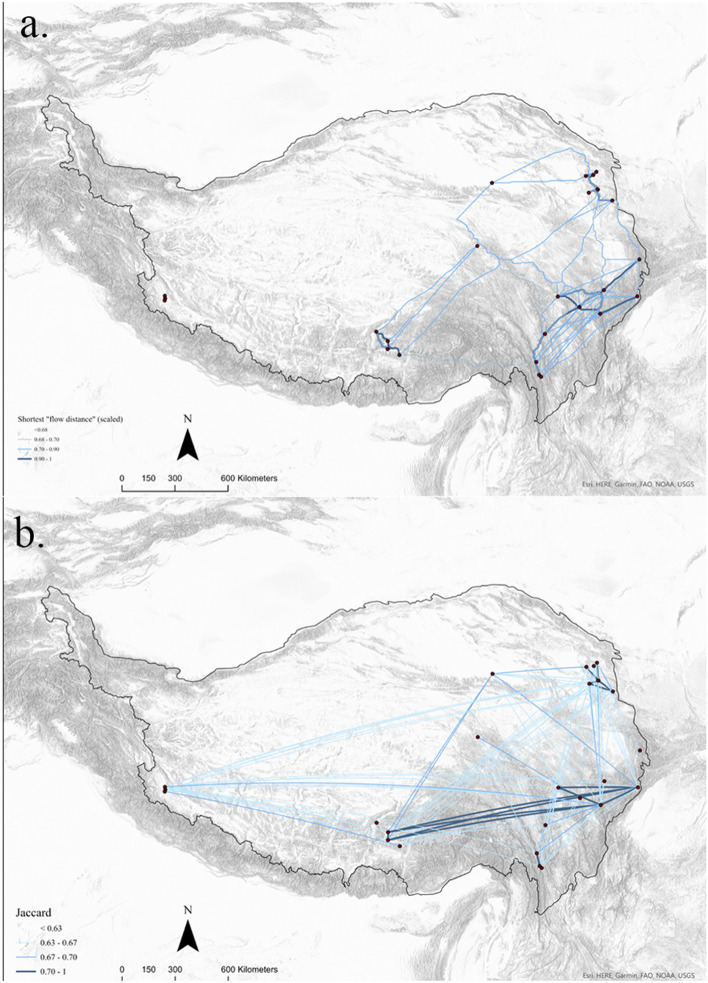
The Subsistence Interactions Network (SIN; **a**) and the ceramic network (**b**). The networks were binarized using arbitrary cutoff values (cutoff value for the SIN: 0.68; cutoff value for the ceramic network: 0.63). The SIN (**a**) visualises modelled the subsistence-focused connectivity for 26 archaeological sites and the ceramic network (**b**) visualises the archaeological cultural connectivity. This map shows that cultural connections correlate with subsistence-focused interactions in the eastern and central plateau regions but not strongly for Western Tibet, which still maintains social ties nonetheless.

The resulting SIN illustrates how real archaeological sites might be connected by the simulated pathways of the SIMM. The lines are weighted by the simulated “flow distance” among sites. The denser the web of pathways and larger flow value a site or a region has, the more prominent the site or region is in the famer-herder interaction network. Well-connected sites or regions tend to have more social capital and geographical convenience to interact (Fig. 5a)^24^. As seen from Fig. 5a, the sites in Central Tibet, Northeastern Tibet and Eastern Tibet are closely connected to each other by simulated pathways. Unsurprisingly, the most substantial ties are present among the geographically closest sites. However, some intriguing geographical patterns emerge. Predominant corridors emerged in Eastern Tibet and Central Tibet. The corridors are connected to the northeastern rim of the Tibetan Plateau either through the Hengduan mountains and northwestern Sichuan grasslands or through the vast grasslands located in Northern Tibet and Southern Qinghai. The northwestern Yunnan sites are loosely connected to the most significant cluster of interactions to their north, channeled by the pathways through the Hengduan Mountains. However, the magnitude of this Yunnan-Sichuan connection is much smaller. Of note, the Western Tibet sites are relatively isolated, and they lack strong ties to reach Central or Northeastern Tibet to participate in the dense geographical networks in the eastern Tibetan Plateau.

### The ceramic network and comparisons between networks

Have the simulated networks of subsistence interactions actually facilitated social interactions in the Bronze and Iron Age Tibet? We investigate this question by constructing a comparative, ceramic-based social network of the same 26 sites previously used in the SIN. The ceramic network broadly quantifies the observed intensity of social interactions in different regions of Tibet. Ceramics are the most abundant material in archaeological sites. The stylistic and technical characteristics of ceramics are often used as proxies for shared community of practice and social signals that indicate certain social or cultural boundaries^8,25,26^. Of note, we recognize that ancient humans communicated with social signals embodied in a variety of material remains beyond ceramics and the sociocultural boundaries materialized in those remains may overlap or significantly vary across space and time^27^. However, here we consider that ceramic morphology is the only and possibly most reliable measurement available in prehistoric Tibet to quantitatively assess cultural interactions and social signals. We also compensate for the limitations of quantitative ceramic network analysis by the qualitative observations of material similarity (including but not limited to ancient ceramics) in Tibet in the Discussion section.

In archaeological social network analysis, the presence and absence of certain morphological attributes of ceramics are also usually used as broad indicators of site similarities and cultural connections^28,29^. We measure the social ties among 26 archaeological sites using a similarity index (the Jaccard Index)^30^. The social network uses archaeological ceramics from 26 sites based on 51 morphological attributes including types of surface treatment, paint, motif and shape (for the calculation of the Jaccard Index see Methods section). When a shared attribute is present in ceramic assemblages, we consider there is a social connection between the sites (in its broadest sense)^28^. The ceramic network is visualized using an arbitrary threshold value and assessed with tie strength and three centrality measurements: degree centrality, eigenvector centrality and betweenness centrality, which indicates how connected a site or region is in the network (for a detailed explanation of those centrality metrics see Supplementary Methods Sect. 4). The comparisons between the SIN and the ceramic social network demonstrate the degree to which the “mobility pathways” from the simulated subsistence interactions were used to facilitate ceramic-based social interactions.

The ceramic network indicates the geospatial arrangement of ceramic similarities as quantified in archaeological data. Sites and regions of the most prominence in trans-regional interactions are bonded by a dense web of social ties. Weak and isolated ties may also indicate notable bridging position, which connects people with different cultural identities^8,24^. As seen from Fig. 5b, the structure of the ceramic social network in the eastern part of the Tibetan Plateau is similar to that of the SIN. The sites in Eastern Tibet are still active agents in channeling interregional movements and probably serve as brokers in the long-distance exchange of ideas, goods, and information. The Northeastern Tibetan site also lies on numerous social ties extending to Central Tibet, Eastern Tibet and Western Tibet, meaning that this region also serves to integrate social groups with different ceramic technologies across the Tibetan Plateau. Ceramics in the Central Tibetan sites are more culturally linked to Eastern Tibet than the rest of the areas, suggesting that Eastern Tibet is an important nexus that facilitated social interactions between Central and Northeastern Tibet. Of note, we observe a significant increase in all three centrality measurements and the value of average tie strength of the Western Tibetan sites in the ceramic social network (see Supplementary Methods Sect. 4 and Supplementary Figures 5 and 6.) This leads to more social connections between Western Tibet and other parts of the Tibetan Plateau, despite a lack of geographical ties in Western Tibet as calculated in the SIN.

The integration of the Western Tibetan sites is the major difference between the ceramic network and the SIN, while the network properties of the rest of the Tibetan Plateau remain largely similar. The network structure demonstrated through the SIMM and quantitative network analysis of the SIN and ceramic social networks bears striking parallels with the cultural landscapes concluded from the existing typological analyses of archaeological artifacts, especially for the dense networks in the eastern Tibetan Plateau^31^. Our results indicate that interpreting social interactions solely with the modelled mobility between farmers and herders in Tibet tends to underestimate the cultural importance of Western Tibet.

## Discussion

The study of farming and herding and the impact of the changing subsistence patterns are among the most important academic inquiries in Tibetan archaeology today. Increasing archaeological research provides evidence of the co-existence of farming and herding among other subsistence modes in Bronze and Iron Age Tibet. Scholars have previously speculated on the possible existence of “semi-nomadic groups” or “agropastoral groups” in several archaeological cultures on the Tibetan Plateau, including the Kayue Culture^32^, Nuomuhong Culture^4^, the stone-cist graves in Western Sichuan^33^ and Western and Northern Tibet^34^. Recent archaeobotanical and zooarchaeological evidence suggested that most of the ancient people on the Tibetan Plateau practiced agriculture to varying degrees in prehistoric times^35,36^. The predominant crops included millet and barley. Recently, there has been considerable momentum in understanding the diverse subsistence strategies across the plateau where hybridization between farming and other forms of food production is commonly documented. Our research reveals a nuanced pattern of associated human mobility and interactions with a clear east-and-west difference.

We further interpret the similarities and differences among the SIMM, SIN and ceramic network based on the results from the typological analysis of archaeological artifacts. Geospatial networks and social network analysis in archaeology are usually used as provocative representations of relationships between entities^25^. In archaeology, connections between those entities are usually established based on subjectively defined similarities. Both the benefits and shortcomings of this approach are due to its quantitative nature. The properties of networks are further complicated by the way in which the observed similarity is interpreted archaeologically. Discrepancies usually exist among the comparisons of the quantitative similarity, the subjective observation of the similarity and the archaeological interpretation of the similarity^37,38^. In this context, the typological analysis of ceramics, as well as stone and bronze artifacts in the past few decades in Tibetan archaeology provide a pertinent source for the assessment and interpretation of the results presented above.

The SIMM, SIN and ceramic social network all point to a common pattern of intensive connections in the eastern part of the Tibetan Plateau. The archaeological evidence in Tibet of the last four decades serves as a qualitative evaluation of our models. The importance of the eastern and northeastern Tibetan Plateau in facilitating trans-regional interactions in prehistory is archaeologically well-known. Tong^39^ noted, for the first time, the similarities between the stone-cist burials and the cultures from the northeastern Plateau and named this pattern the “Crescent-shaped Cultural Communication Belt”. Further archaeological research in recent decades indicated broad-scale similarities in different types of material remains, including ceramics, bronze weapons, ornaments and burial traditions^31^. The latitudinal connectivity along the eastern rim of Tibet may have been rooted in the Neolithic Age, evident in the ceramic analysis of the Majiayao Culture^40^. The latitudinal connectivity was much more intensive and geographically extensive during the fourth millennium and third millennium BP^33^.

Central Tibet (Lhasa and its surroundings) is a relatively independent cultural region in the Late Bronze and Early Iron Ages, with weak cultural connections to Western Tibet and Eastern Tibet. The relatively independent nature of the Qugong Culture (ca. 3800–3000 BP) in the fourth millennium BP is inherited by the remains in Bangga, dated between ca. 3000–2200 BP. The material assemblage at Bangga revealed that the difference of cultural artifacts between the Qugong Culture and Bangga is an endogenous cultural change according to our previous research^12^. The connections between Central Tibet and Eastern Tibet are evident in shared traditions of polished surface treatment and decorations in ceramics, forms of stone-cist burials, millet cultivation and pig rearing, although such shared traditions are unambiguous in some regions than others^41,42^. We also see cultural connections between Eastern Tibet and Central Tibet from the typological analysis of bronze mirrors, where the Eastern Tibetan, Central Asian and local style mirrors coexisted in the third millennium BP^43^, although the nature and pathways to such cultural cohesion are not yet clear.

The SIMM, SIN and ceramic network capture, to a reasonable degree, this interconnectivity among communities in the northeastern, eastern and central Tibetan Plateau. All our models show that the pathways and cultural connections in the eastern rim of Tibet prevail, indicating that the modelled subsistence interaction between farmers and herders correlates with the pattern of social interactions as seen in the ceramic network in those parts of Tibet.

Why is Western Tibet still culturally connected to the rest of the Plateau in the ceramic network despite its lack of geographical ties in the SIMM and SIN? A possible interpretation of the modelled east/west difference is that the social interactions between Western Tibet and other areas in Tibet were not facilitated by the subsistence mobility “highways” within the Tibetan Plateau, but were shaped by the cultural participants outside of the Tibetan Plateau. Archaeologists argued that the cultural influences from outside of the Tibetan Plateau had played a significant role in shaping the pattern of social interactions in Tibet in the fourth and third millennium BP^42,44^. Recent investigations focusing on ceramics unearthed from burials in the Phiyang-Dungkar in Zanda County, dated to the third millennium BP, demonstrate significant typological similarities of ceramics with those previously uncovered in the Mustang region, Nepal^45,46^. We also observe a widespread practice of using cord decoration, usually on red and orange unburnished ceramics, among sites in the broader western and southern Himalayan regions in the third millennium BP. The black-burnished ceramics during the fourth millennium BP in Kashmir, including Burzahom, Gurkral and Kanispur, among others, are also similar to those from Qugong in Central Tibet (Fig. 1)^47,48^. This raised an awareness that the materials from Kashmir could play a role in bridging the gap between Western and Central Tibet through a trans-Himalayan movement that has yet been fully investigated. During the third millennium BP, increasing evidence suggested trans-regional cultural communications. The common typology of spouted jars in Central, Western Tibet and Xinjiang indicates another pathway of cultural influence connecting Tibet with territories beyond the plateau, including Xinjiang^45^. Recent archaeological research also suggested that a variety of material remains were exchanged between the southern periphery of the Central Asian mountains and the Himalayan regions^49–51^.

We speculate that the trans-Himalayan cultural exchanges between Tibet and regions beyond the Plateau (the Kashmir region in particular) may have contributed to the discrepancies between the ceramic network and the SIN (Supplementary Figs. 5–6; Supplementary Table 2–3). The ancient populations from the western and southern frontier of the Himalayas may have formed a variety of social, commercial and cultural connections with the people living on the Tibetan Plateau through trans-Himalayan farming-herding interactions. Direct pastoral movements and trade across the Himalayas was common, according to ethnographical records^52^. It is less likely that the connection between Western and Central Tibet and Xinjiang is bridged by massive direct movements across the desolate area of the Changtang steppe in Northern Tibet since there are few documented human settlements to serve as logistic locations on those routes. Human movements to the south and west of the Tibetan Plateau, especially the trans-Himalayan movements, likely account for the social ties between Western Tibet and Central Tibet as seen in the ceramic network. People in Western Tibet in the fourth and third millennium BP possibly did not participate directly in the dense subsistence interaction networks within the eastern Tibetan Plateau. The trans-Himalayan human movements, as seen in the typological analysis of archaeological artifacts, may have changed the network structures of cultural interactions on the Tibetan Plateau during the Bronze and Iron Ages.

Climate change through the Holocene presents challenges to accurately model past landcover using modern remote sensing data. In this research, we derived vegetation proxy data from medium-resolution modern satellite imagery for use in modeling mobility (flow) and interactions across the Tibetan Plateau. Published reconstructions of the precipitation and temperature on the Tibetan Plateau based on 38 fossil sporopollen records indicate that the precipitation and temperature remained stable and resembled the modern climate during the Late Holocene (after 4.4 kaBP), except for a significant cooling period around 2.1 kaBP^53,54^. The amplitude of variation of the pollen-based Holocene temperature reconstruction is less than 1.5 °C^55^. Possibly owing to the weakening of East Asian summer monsoon and/or the emergence of pastoralism^56–58^, the extent of alpine steppe and alpine meadow on the Tibetan Plateau vacillated variously during the Late Holocene^59^. Chen and colleagues synthesized the *Artemisia*/Cyperaceae ratios of five pollen records from near the boundary of alpine meadow and steppe, suggesting a stable extent of alpine steppe/desert and an extension of alpine steppe/meadow around 1.0 kaBP^55^. However, the magnitude of grassland landcover change is also small during the Late Holocene. Herzschuh modelled the vegetation cover on the Tibetan Plateau and found that Tibetan vegetation is sensitive to changes in CO_2_^59^. Antarctic ice core records suggested that the atmospheric CO_2_ concentration during 3600–2000 BP is around 280 ppm^60^, which is significantly lower than present-day conditions in Tibet (above 375 ppm).

Given these reconstructions, and since the increasing CO_2_ level during Holocene suggests a general trend of shrinking low-biomass and drought-resistant vegetation (e.g., alpine steppe) through time^59^, we expect that the extent of grassland from 3600 to 2200BP might be similar or slightly larger than that of the present day. Therefore, our models tend to underestimate the extent of farmer–herder interactions in the Bronze and Iron Ages, since high-productivity vegetation contributes the most to our modelled mobility flows. Despite our potential underestimation of the herding range of mobility, the statistically significant correlation between the SIMM and known archaeological sites suggests that centuries of settlement by ancient agropastoralists reflects a similar distribution pattern as high-productivity vegetation today, suggesting positive feedback in shaping the long-term geography of cultural interactions across Tibet, perhaps even more than our model can illustrate.

Although the Himalayan regions and the southern and eastern peripheries of the broader Inner Asian Mountain Corridor regions, mainly Kashmir and Xinjiang, may have actively participated in the shaping of complex interaction networks in Tibet, other potential interpretations of the differentiated patterns of farmer and herder interactions and social interactions in Tibet remain. For example, the archaeology on the southern rim of the Himalayas is still very limited. It is possible that ancient trans-Himalayan movements among Nepal, Bhutan and Tibet also play a major role in facilitating the patterns of social interactions in Tibet^42^. Due to the lack of quantitative archaeological data, we also did not consider the emerging social complexity in shaping the patterns of human mobility and social interactions in our model. Historical documents and increasing archaeological evidence of prestige goods circulation indicate the possible emergence of elite classes and local political centres in this period^61^. Although large settlements and citadels of regional political centres in the Bronze and Iron Ages not been discovered in Tibet so far, recent discovery of high-ranking burials in Western Tibet suggest that socially stratified regional polities may also underpin the exchange networks of a variety of goods as early as around 3000 BP^62^.

## Conclusion

Though a difficult terrain to travel, the Tibetan Plateau has been a hub of cultural connectivity and social cohesion since prehistoric times. The increasing cultural interactions and the rise of herding and farming in Tibet are not merely regional phenomena but also intertwined with processes on a broader continental scale^63^. This research contributes to this line of scholarly inquiry by presenting quantitative models that interpret the cultural diversities and social interactions with a geospatial framework of subsistence interactions between farmers and herders. We draw attention to social interactions in the Bronze and Iron Age Tibet that were at least correlated with two factors: (1) the geography of the interactions between farmers and herders (modelled as aggregated high flow accumulations between pastures and arable lands), and (2) the external cultural influences between sites on the western Tibetan Plateau and beyond. The results provide insights into a more nuanced interpretation of the relationships between subsistence economy and social interaction.

The modelling approach in this study has, of course, some technological and interpretative limitations. This study used modern vegetation data as a proxy for past landcover in the Bronze and Iron Ages, the temporal-spatial accuracy of which is coarse and provides only a general picture of complex vegetative dynamics through time. Whatsmore, the subsistence strategies among ancient populations of the Tibetan Plateau became greatly diversified from the 3rd to 2nd millennia BC after the increase introduction and adaptation of exotic domesticated animals and crops, such as millet, barley, sheep, goat and cattle^3^. Our model presents a general picture of herding mobility, when in actuality different animals require specific strategies related to altitude, seasonality etc. Yet in spite of these nuances, our model indicates that interactions formed among farmers and herders, broadly conceived as specialized sectors of a joint economy^64^, were foundational in shaping the observed cultural changes and settlement patterns throughout the Bronze and Iron Ages. Our results further reproduce the archaeologically observed east/west difference in the patterns of subsistence interaction and social interactions, where Western Tibet is relatively isolated and the rest of the Tibetan Plateau is more socially and geographically interconnected.

This result resonates with the recent debates on the ancient genomic history of Tibet and the origin of the Sino-Tibetan language family^65–67^. Recent genomic analysis has revealed that the ancestry of northeastern and central-southern Tibet communities is more internally clustered, whereas ancient Western Tibetan populations share more alleles with Central Asian individuals^66^. Meta-analysis of ancient and modern genome-wide data also suggest that central, eastern, and northeastern Tibetan share the strongest genetic affinity with their geographical neighbours^68^. The east/west difference as modelled in this research broadly corresponds with these conclusions, indicating that intensive cultural interactions on the eastern rim of Tibet may have played an important role in shaping these patterns of genetic affinity. However, our study further suggests that the shared cultural identity and geography of genetic ancestry across the entire Tibetan Plateau reflects nuanced regional conditions that emerged from longstanding patterns of social interactions and human mobility, specifically where Western Tibet is significantly different from those in the east, possibly owing to outside cultural participants.

## Methods

### The subsistence interaction mobility model (SIMM)

To understand the optimal pathways of the mobility of farming-herding interactions, we developed the Subsistence Interaction Mobility Model (SIMM) that simulates human mobility from all cells across the study area toward defined areas of arable lands. Those simulated movements are weighted to prioritize flow over cells categorized according to vegetation. We used modern satellite imagery to calculate the Normalized Difference Vegetation Index (NDVI), which is used as a proxy for the productivity of vegetation. NDVI is an index measuring the greenness of vegetation, which is calculated from the multispectral satellite images based on the formula below:

$${\text{NDVI}} = \left( {{\text{NIR}} - {\text{RED}}} \right)/\left( {{\text{NIR}} + {\text{RED}}} \right)$$

where RED and NIR stand for the spectral reflectance measurements acquired in the red and near-infrared ranges, respectively.

The SIMM model uses the flow accumulation method developed by Frachetti et al.^17^ which simulates human movement as accumulated flow across a cost surface, where greener vegetation is classified as comparatively less costly. This approach calculates accumulated flow value as the sum value of all cells flowing into each neighboring cell. In our application of the method, each iteration of the model simulates flows that are spatially oriented to accumulate from all non-cropland cells toward a single patch of arable land (prioritizing the greenest vegetation) and then iterated for all patches. After aggregating the rasterized flow values of each cell from all iterations, we illustrate the optimal, high-vegetal pathways over which farmers and herders might interact. In this model, the resulting map (Fig. 3) consists of raster cells with different accumulative flow values, indicating the simulated amount of movement or ‘traffic’. We visualized the most frequently traversed pathways based on a cutoff value above one standard deviation. The raster cells below this cutoff value are transparent. After applying the cutoff value, the red lines on the map demonstrate the “mobility highways” where the hypothesized subsistence interaction mostly occurred. A recent Least Cost Paths (LCP) analysis considering Tibetan archaeology applied a different method to our approach here. Lancuo and colleagues modelled the networks of Neolithic and Bronze Ages sites across different modern landcover and correlated the networks with archaeobotanical and zooarchaeological evidence^14^. Methodologically, LCP approaches use the location of sites as a nodes in modelling path of connectivity, whereas the flow accumulation method does not require archaeological sites as input data, hence allowing these data to be used for model testing in archaeological geospatial analysis.

### Evaluation of the SIMM

Following the method applied by Frachetti and colleagues^17^, we evaluated the performance of the SIMM by comparing the Z-scores of the mean flow value and distance to simulated pathways between 1434 archaeological sites and 500 cohorts of randomly generated points. The Z-score is calculated as:

$${\text{z}} = \left( {{\text{x}} -\upmu } \right)/\upsigma ,$$

where x is the value of archaeological sites, μ is the mean of random points, and σ is the population standard deviation of random points.

The Z-scores measure how many standard deviations the mean values of archaeological points are below or above those of random points. We also test the statistical difference of the mean flow value and distance to pathways using one-tailed Student’s t-test and the results are both significant.

### The subsistence interaction network (SIN)

To develop a measurement of the conceptual “flow distance” among archaeological sites based on the simulated mobility “highways”, we converted the SIMM into a formal network, the SIN. Instead of measuring the interconnectivity of archaeological sites using geographical distances, we calculated the least-cost pathways among sites based on the cost surface of the inverted flow values in the SIMM using the “Least-Cost Path as Distance” function in ArcGIS Pro (Supplementary Methods Sect. 2). In SIN, the higher the value of the raster cell (the scaled and inverted flow values), the higher the travel resistance. Based on this method, the geographical connectivity of the 26 well-excavated sites is measured by the shortest “flow distance”, assuming that the simulated mobility “highways” are used to facilitate interactions among sites. The shortest “flow distance” are scaled between 0 and 1 and visualized with binary cutoff values.

### The ceramic network

To investigate how the simulated subsistence interaction correlates with the social interactions observed in archaeology, we used ceramic data from 26 excavated sites in Bronze and Iron Age Tibet to construct the social network. The ceramic data were compiled from excavation reports and monographs. The network ties were constructed using the Jaccard index^30^, based on the presence or absence of 51 morphological attributes of ceramics. The Jaccard similarity between sites is calculated using the formula below:

$${\text{Jaccard}} = {\text{a}}/\left( {{\text{a}} + {\text{b}} + {\text{c}}} \right)$$

where (a) is the number of attributes of ceramics shared in both sites; (b) is the number of attributes presented only in the first ceramic assemblage; (c) is the number of attributes presented only in the second ceramic assemblage. The Jaccard index is a value ranging from 0 to 1. The higher the Jaccard index, the higher the similarity (see Supplementary Method Sect. 3).

We evaluated the network property of each site and region using tie strength and three centrality measurements: degree centrality, eigenvector centrality, and betweenness centrality, which are commonly used in social network analysis^69^. Although the network graphs were visualized using an arbitrary cutoff value as the method permits, we tested the robustness of the centrality measurements of different regions in R, based on the ranks of centrality measurements under different cutoff values (Supplementary Methods Sect. 4).

### Supplementary Information

Supplementary Information 1.

Supplementary Information 2.

Supplementary Information 3.

Supplementary Information 4.

Supplementary Information 5.

Supplementary Information 6.

Supplementary Information 7.

Supplementary Information 8.

## Data Availability

The datasets generated and analysed during the current study are available in GitHub (https://github.com/XinzhouChen/SIMM-SIN-and-ceramic-network--raw-data-and-tables).

## Code availability

All Python and R scripts used to generate and evaluate the models in this study and a user instruction file have been deposited in GitHub (https://github.com/XinzhouChen/SIMM-SIN-and-ceramic-network--raw-data-and-tables).
