# Supplementary material for: Geospatial modelling of farmer–herder interactions maps cultural geography of Bronze and Iron Age Tibet, 3600–2200 BP
Source: Sci Rep. 2024 Feb 2;14:2010. doi: 10.1038/s41598-023-50556-9 (PMC10837149; doi:10.1038/s41598-023-50556-9)
Supplement: Supplementary file 1 — Supplementary Information 1. [file 41598_2023_50556_MOESM1_ESM.docx]

**Supporting Information for**

Geospatial modelling of farmer-herder interactions maps cultural geography of Bronze and Iron Age Tibet, 3600-2200 BP

Xinzhou Chen*^1,2^, Hongliang Lü*^2^, Xinyi Liu^2^, Michael D. Frachetti*^2,3^

Corresponding authors: xinzhouchen@wustl.edu**;** luhl@scu.edu.cn**;** [frachetti@wustl.edu](mailto:frachetti@wustl.edu)

^1^ Center for Archaeological Sciences, Sichuan University, Chengdu, Sichuan Province, China.

^2^ Department of Anthropology, Washington University in St. Louis, St. Louis, MO, United States of America.

^3^ School of Cultural Heritage, Northwest University Xi’an China

The following supporting information provides: 1) the methodological details and paleoenvironmental considerations of constructing and evaluating the Subsistence Interaction Mobility Model (SIMM); 2) the methodological details of converting SIMM to a formal network, the Subsistence Interaction Network (SIN); 3) the methodological details of constructing the ceramic social network. 4) the methodological details of evaluating the SIMM, SIN and ceramic social network.

The Python and R scripts and archaeological data we developed and collected to construct the SIMM, SIN and ceramic social network were deposited in the GitHub repository (https://github.com/XinzhouChen/SIMM-SIN-and-ceramic-network--raw-data-and-tables).

**Supplementary Methods**

**Section 1. The Subsistence Interaction Mobility Model (SIMM)**

- 1. The flow accumulation method to construct the SIMM

In archaeology, mapping optimal pathways is usually based on the method of least-cost path (LCP) and the other variants of LCP. LCP is a method connecting several locations by generating a path with the least accumulative, user-defined cost. In archaeology, this method is normally based on Dijkstra’s algorithm and can be easily performed in most of the current geospatial analysis software (e.g., GRASS, ArcGIS, QGIS, etc.). A standard least-cost path analysis requires three elements: origin, cost, and destination. A variety of cost functions for predicting human movement have been applied successfully in archaeological research, including Tobler’s hiking function, Pandolf’s function, and Bell and Lock’s function, etc.^1,2^

Despite the advantages of LCP analysis, one of the drawbacks of this approach is that it often requires a known destination or origin. Most of the built-in functions in contemporary geospatial analysis software do not permit the generation of pathways without either origin or destination, which are usually unknown when simulating past movements. Several attempts have been made to solve this problem. One workaround is producing multiple pathways connecting multiple origins and/or destinations to generate the least-cost networks of human movement^3,4^. White and Barber^5^ attempted to overcome the one-to-one pathways by introducing the “From Everywhere to Everywhere” (FETE) approach. This method calculates the LCPs between every point and its neighbours iteratively, resulting in the generation of least-cost corridors without assuming either destination or origins. However, the FETE method is very computation-intensive and time-consuming, especially when using a high-resolution cost raster covering a large area^6^. A less computation-intensive way of generating least-cost networks is based on hydrological analysis toolkits^7,8^.

The modelling method we used here is a combination of the FETE and the flow accumulation method^9^. This approach calculates accumulated flow value as the accumulated weight of all cells flowing into each downslope (higher NDVI cells, in our case) cell. The flow accumulation model, like standard LCP analysis, generates a raster map that indicates pasture-based optimal pathways on a friction surface (NDVI in this research, see Section 1.3) without pre-determined origins or destinations. For the complete Python program of this model see the Python code in the GitHub repository and Supplementary Figure 1.

- 1. Paleoenvironmental considerations

Large-scale human movements and cultural and subsistence changes on the Tibetan Plateau are often associated with climate changes^10,11^. Chen and colleagues^10^ argued that the major human movements onto the Tibetan Plateau were associated with the warm and humid climate after the Last Deglacial period (18-11.6 ka BP), based on the reconstruction of the East Asian Monsoon and Indian Summer Monsoon^12^. The rise of agropastoralism in Tibet is associated with the cooling climate after the fourth millennium BP^13^.

In this research, we use medium-resolution modern satellite imageries to model the farmer and herder interactions across the Tibetan Plateau. Large-scale reconstructions of the precipitation and temperature on the Tibetan Plateau indicate that the climate remains stable and resembles the modern climate after the end of the Holocene Maximum (around 4400 BP)^14,15^. Although climate change may act as a factor that contributes to cultural change, human agency and resilience are also fundamental considerations when matching paleoenvironmental records with variations of ancient human activities. Therefore, we consider that it is acceptable to model large-scale human activities using modern environmental proxies based on three reasons: 1) The spatial and chronological resolution of ancient climate change reconstruction is too coarse to simulate large-scale human activities through time; 2) Previous research suggested that the climate after the mid-late Holocene did not change in such a great magnitude that drastically influenced the geospatial pattern of human activities in Bronze and Iron Age Tibet; 3) Sociopolitical factors and human agency in high-elevational environments are yet to be investigated in archaeology. The way in which sociopolitical organizations of human society and culture connectivity may have responded to climate change through time are better-discussed case by case qualitatively instead of being incorporated in the quantitative models in this research.

- 1. Constructing the SIMM

We kept the research area within the Tibetan Plateau for simplicity. The extent of the Tibetan Plateau used in this research is defined by Zhang and colleagues^16^. The shape file used in the model was downloaded from the database of the National Tibetan Plateau and Third Pole Environmental Data Center^16^.

We downloaded the modern land cover data provided by the Food and Agriculture Organization of the United Nations^17^. The cropland pixels were vectorized to points using the “Raster to Points” function in ArcGIS and used as the origin points in the flow accumulation model. The vectorized points represent a rough estimate of the locations of arable lands on the Tibetan Plateau. We used the Normalized Difference Vegetation Index (NDVI) values as the cost surface in the flow accumulation model. NDVI is an index measuring the healthiness of vegetation, which is calculated from the multispectral satellite images based on the formula below:

. NDVI = (NIR - RED)/ (NIR + RED)

where RED and NIR stand for the spectral reflectance measurements acquired in the red and near-infrared regions, respectively. The NDVI raster is downloaded from the United States Geological Survey Data Archive, in the EROS Moderate Resolution Imaging Spectroradiometer (eMODIS) dataset^18^. The NDVI raster in our model represents the modern vegetation of the Tibetan Plateau in August 2020, which serves as a rough estimate of the vegetation in ancient times (Supplementary Figure 2). We chose to use the NDVI raster in August since it is the season when human movements, pastoral activities and vegetation productivity are all at their prime in Tibet. The cell size of the NDVI raster is 250 meters, meaning that the width of every cell in the raster is 250 meters and vegetal variations below that threshold are not recognizable. NDVI is a value ranging from -1 to 1, where values approaching -1 indicate water bodies, rocks, and barren land. The original NDVI is scaled from 0 to 1.

Over each iteration, we ran the flow accumulation of movements towards a single cropland point (Supplementary Figure 3: a), producing a raster map representing all the possible pathways moving to this agricultural settlement along the optimal routes of high vegetal patches within the Tibetan Plateau. After 6459 iterations (number of iterations = the total number of vectorized croplands), we summed all the raster and produced a new raster map indicating the aggregate flows of subsistence interactions where the most frequently travelled “mobility highways” are highlighted with extremely large raster values (Supplementary Figure 3: b). The raster values on the map indicate the traffic volumes in the hypothesized interactions between herders and farmers. Although the result of the SIMM is displayed using an arbitrary cutoff value of a standard deviation above the mean, the statistical evaluation of the model using archaeological site locations presented below uses the actual floating-point values in the resultant raster.

1.4 Evaluating the SIMM with locations of archaeological sites.

To evaluate the SIMM with known locations of archaeological sites. We compiled a dataset including 1434 archaeological sites dated between 3600 BP and 2200 BP (see Dataset 1 in the GitHub repository). Most of the sites in this dataset are from legacy archaeological surveys in the past few decades and are mapped in the Cultural Relic Atlas of China^19,20,21,20,22^. In ArcGIS Pro, we georeferenced the all the Cultural Relic Atlas in Tibet to get the exact location of the sites. Of note, although most of the sites are not directly radiocarbon dated, archaeologists determined the relative dates of sites to cultures/phases (e.g., Shang, Zhou, Kayue, Xindian, etc.) by examining the typology of ceramics. Some sites may have a multi-period occupational history different from the records on the Atlas, as demonstrated by recent archaeological surveys and excavations^23,24^. The chronological control of the dataset is thus relatively poor. However, this is still acceptable for the evaluation of this model, as we consider the sites between 3600 and 2200 BP to be roughly contemporary and represent the general picture of settlement patterns and social interactions after the introduction of agropastoralism on the Tibetan Plateau. The result of the model evaluation is discussed in the main text and is statistically significant as shown in the Student’s t-test (Fig. 3). We further separated all archaeological sites into two groups, sites within and outside of modern croplands, to test if those two types of sites demonstrate different statistical patterns in the model (Supplementary Figure 4). The results indicate that the model is still statistically significant when the model was tested with sites located within and outside of croplands. Of note, sites outside of modern croplands have higher flow values and longer distances to the simulated pathways than those within modern croplands. This result show that sites outside of the modern cropland are generally more traversed in the simulated farming-herding activities, thus they may have higher potential for non-agricultural activities in this mobility network.

**Section 2. The Subsistence Interaction Network (SIN; derived from SIMM)**

The SIMM is a geospatial model constructed based on modern environmental proxies including the NDVI and landcover data. To develop a measurement of the network properties of actual archaeological sites, we converted the SIMM into a formal network, the SIN, that measures the shortest “flow distance” among 26 archaeological sites within the Tibetan Plateau (Supplementary Figure 1: b).

In the SIN, we consider that for each given cell, the higher the flow accumulation value (the modelled traffic volume), the lower the travel resistance. Therefore, the connectivity among any pairs of archaeological sites is calculated as a pathway that passes through the highest accumulative flow value (modelled in the SIMM) between them. We measured the shortest “flow distance” among 26 sites using the “Cost Distance” function in ArcGIS, and the flow accumulation raster map produced in the SIMM was used as the friction surface in the calculation of the shortest “flow distance”.

As shown in Figure 5: a and discussed in the main text, the SIN demonstrates that there are two broad patterns of connectivity in Tibet. Twenty-three sites in the Central, Northeastern and Eastern Plateau form a significant cluster connected by the pathways in Western Sichuan and Southern Qinghai; while three sites in Western Tibet form a small, relatively isolated cluster. To investigate how the interactions between farmers and herders may be correlated with patterns of cultural interactions, we used the SIN to quantitatively compare with the social/cultural interconnectivity measured by the Jaccard Index of ceramic similarities (see Sections 3 and 4).

**Section 3. The ceramic network**

- 1. Preparing data for the social network analysis of archaeological ceramics

We used the presence-and-absence data of the morphological attributes of ceramics as a rough estimate of the social interactions among archaeological sites. Many archaeological network analyses use percentage statistics of ceramic types or the presence and absence of artifact typology as indicators of social ties^25,26,27^, which are different from the approach used here. We consider that it is acceptable and viable to broadly model the social interactions within the Tibetan Plateau in this period with the presence-and-absence data mainly because: 1.) Tibet is relatively understudied in archaeology and a detailed artifact chrono-typology is not available; 2.) The current stylistic analysis in Tibet is mostly based on a qualitative comparison of ceramic forms and decorations, providing us with a robust body of literature that succinctly summarizes how certain morphological attributes of ceramics are associated with cultural identities and social interactions^28^; 3.) Chinese archaeological excavation reports only publish artifacts selectively, so the percentage data of ceramic forms is not available in Tibet. Most excavation reports, however, summarize the presence and absence of ceramic attributes (especially ceramic forms, types of decorations, and surface treatments) based on a relatively uniform standard. Therefore, using the presence-and-absence data of ceramics is a relatively reliable way to characterize the variability of the assemblages in sites and measure the intensity of social interactions and social signalling; 4.) Although constructing social ties with presence-and-absence data falls short of capturing the possible differences shown in the percentage of ceramic attributes, the latter of which may be important indicators of cultural diversities in many cases^29,30^, this approach is better at quantifying vaguer similarities of material assemblages, which has been discussed and applied in previous archaeological social network analyses in other regions^31,32^.

We prepare the ceramic attribute table based on the descriptive paragraphs in Chinese archaeological reports and the visual examination of the pictures and line drawings of ceramics (except for two archaeological sites: Changguogou and Bangga, for which we examined both the archaeological ceramics published in the reports and those curated at Sichuan University, For a complete table of the presence and absence of ceramic attributes Supplementary Table 1 and Dataset 2 deposited in the GitHub repository.

- 1. Constructing the ceramic network

We construct social ties based on the similarities of ceramic attributes using the Jaccard Index. When a shared attribute is present in the ceramic assemblage, we code with the value 1, indicating that there is a correlation between the sites; otherwise, we give a value of 0 between the sites, suggesting no correlation. The Jaccard similarity between sites is calculated using the formula below:

Jaccard = a / (a + b + c)

where (a) is the number of attributes of ceramics shared in both sites; (b) is the number of attributes presented only in the first ceramic assemblage; (c) is the number of attributes presented only in the second ceramic assemblage. The Jaccard index is a value ranging from 0 to 1. The higher the Jaccard index, the higher the similarity (Supplementary Figure 1: c).

**Section 4. Visualizing and evaluating the network structures of the SIN and ceramic network**

The SIN and the ceramic network have different archaeological implications. The SIN, derived from the SIMM, measures the connectivity among sites in the inter-regional exchange of material, goods, and ideas, assuming that ancient people travel along the simulated “mobility highways” as much as possible. Those exchanges mostly happen during trade, everyday pastoral activities, wars, gifting, or other types of connections occurring between farmers and herders. The ceramic social network, derived from the empirical characterization of ceramic forms, represents ceramic-based social and cultural interactions actually observed and quantified archaeologically^26^. Thus, the comparison between those two networks indicates the degree to which the available geographical ties in Tibet are used to facilitate trans-regional interactions in Tibet.

Despite numerous node-level and graph-level measurements that quantify the properties of nodes and whole networks^33,34^, we rely on three centrality scores mostly commonly used in social network analysis and the average tie strength to quantify the network properties of different regions as modelled in the SIN and ceramic network. A central position of certain points, as Freeman points out^34^, indicates the extent to which a point can avoid the control potential of other points in information communication. The centrality of points can be broadly understood as its level of independence^34^. The calculation of all centrality scores was performed in R using the free package “igraph”^35^. Based on a robust body of literature on social network analysis^33,36,37,38^, we briefly explain the calculation and archaeological implications of the three centrality scores:

Degree centrality: degree centrality is the most used centrality score in archaeological networks. Degree centrality is the number of existing ties of a node. In a weighted network, the degree centrality of a node is the sum of its valued ties^35^. Degree centrality measures a node’s exposure in the network. The more ties that the node holds, the more likely this node is exposed to more information and material flows. We calculate the weighted degree centrality using the “strength” argument in R.

Eigenvector centrality: eigenvector centrality is a measurement of centrality in which a node’s centrality is its summed connections to others, weighted by those nodes’ centralities^35^. Therefore, if a node has only limited connections connected to other well-connected nodes, this given node will have a low degree centrality but a high eigenvector centrality. We calculate the weighted eigenvector centrality using the “eigen_centrality” argument in R.

Betweenness centrality: betweenness centrality is defined by the number of shortest paths going through a node^35^. Higher values of betweenness centrality mean more nodes depend on this given site to reach other sites. Nodes with a high betweenness centrality thus indicate that this site owns more social capital for acting as a broker that controls the information and material flows in the network. We calculate this score using the “betweenness” argument in R.

The calculation and comparison of the centrality scores among sites depend on the way in which the ties (measured by floating-point numbers) are binarized. Different threshold values that dictate whether a social tie exists among archaeological sites will result in different structures of the social network. We visualized the SIN and ceramic network with arbitrary cutoff values (0.68 for the SIN; 0.63 for the ceramic network). Those threshold values were chosen so that both networks are visualized with a similar number of ties after applying the threshold values (242 ties in the SIN; 244 ties in the ceramic network)^30^. The summary of the weighted centrality scores of the two networks under these threshold values is shown in Supplementary Table 2 and 3.

In social network analysis in archaeology, the threshold value to determine whether the ceramic assemblage of a site is significantly similar to another site is usually arbitrarily defined^26,38^. To further validate the role of different regions in this modelled pattern of social and subsistence interaction, we iteratively calculated the weighted centrality scores under all possible threshold values. Following the approach by Roberts and colleagues^39^, we compare the average ranks of the three centrality scores and tie strength among different regions. The results are shown in Supplementary Figures 5 and 6. In the statistical comparison of the ranks of centrality metrics and tie strength, the higher the ranks and tie strength, the more prominent the site is in the overall network structure. Those sites may have higher social capital and geographical convenience to interact with other sites.

As discussed in the main text, the SIN and ceramic network demonstrated different patterns of interactions in the eastern and western parts of the Tibetan Plateau. The visualization of the network with arbitrary cutoffs suggests that the subsistence interactions of Northeastern and Eastern Tibet facilitated the social interactions in those regions, as evidenced by the dense connections present in the east of the graphs. The iterative calculation of the three centrality scores further validates this pattern as Eastern and Northeastern Tibet have higher ranks of centrality scores and tie strength than Central and Western Tibet. The tie strength and the ranks of the centrality scores of Western Tibet are the lowest among all regions, suggesting that the farmer and herder interactions are not intensive in Western Tibet. However, in the ceramic network, the tie strength and betweenness centrality of Western Tibet drastically increases. Based on qualitative analysis of the similarities of archaeological artifacts between Tibet and its surrounding regions presented in the Main Text, we speculate that this pattern of subsistence and cultural interactions in Tibet is likely associated with the external influences from Kashmir. Kashmir’s participation in the movement and material exchange network in Tibet, especially in Western and Central Tibet, may lead to the simulated network structures of interactions in Tibet.

**References**

1. Herzog, I. A review of case studies in archaeological least-cost analysis. *Archeol. E Calcolatori* **25**, 223–239 (2014).

2. Verhagen, P., Nuninger, L. & Groenhuijzen, M. R. Modelling of pathways and movement networks in archaeology: an overview of current approaches. in *Finding the Limits of the Limes* 217–249 (Springer, Cham, 2019).

3. Howey, M. C. Using multi-criteria cost surface analysis to explore past regional landscapes: a case study of ritual activity and social interaction in Michigan, AD 1200–1600. *J. Archaeol. Sci.* **34**, 1830–1846 (2007).

4. Bell, T., Wilson, A. & Wickham, A. Tracking the Samnites: landscape and communications routes in the Sangro Valley, Italy. *Am. J. Archaeol.* **106**, 169–186 (2002).

5. White, D. A. & Barber, S. B. Geospatial modeling of pedestrian transportation networks: a case study from precolumbian Oaxaca, Mexico. *J. Archaeol. Sci.* **39**, 2684–2696 (2012).

6. Crabtree, S. A. *et al.* Landscape rules predict optimal superhighways for the first peopling of Sahul. *Nat. Hum. Behav.* **5**, 1303–1313 (2021).

7. Frachetti, M. Digital archaeology and the scalar structure of pastoral landscapes. in *Digital archaeology: bridging method and theory* 128 (Routledge, 2005).

8. Fábrega-Álvarez, P. & Parcero-Oubiña, C. Proposals for an archaeological analysis of pathways and movement. *Archeol. E Calcolatori* **18**, 121–140 (2007).

9. Frachetti, M. D., Smith, C. E., Traub, C. M. & Williams, T. Nomadic ecology shaped the highland geography of Asia’s Silk Roads. *Nature* **543**, 193–198 (2017).

10. Chen, F. H. *et al.* Agriculture facilitated permanent human occupation of the Tibetan Plateau after 3600 BP. *Science* **347**, 248–250 (2015).

11. Dong, G. *et al.* Dispersal of crop-livestock and geographical-temporal variation of subsistence along the Steppe and Silk Roads across Eurasia in prehistory. *Sci. China Earth Sci.* **65**, 1187–1210 (2022).

12. Dykoski, C. A. *et al.* A high-resolution, absolute-dated Holocene and deglacial Asian monsoon record from Dongge Cave, China. *Earth Planet. Sci. Lett.* **233**, 71–86 (2005).

13. Zhang, D. *et al.* Shiqian renlei xiang qingzanggaoyuan kuosan de lishiguocheng he keneng qudongjizhi. *Zhongguokexue Diqiukexue* **8**, 1007–1023 (2016).

14. Hou, G., E, C. & Xiao, J. Qingzanggaoyuan quanxinshi jiangshui xulie de jichengchongjian. *Prog. Geogr.* **31**, 1117–1123 (2012).

15. Li, F., Hou, G., E, C. & Jiang, Y. Qingzang gaoyuan quanxinshi qiwen xulie de jichengchongjian. *Arid Zone Res.* **32**, 716–725 (2015).

16. Zhang, Y., Li, B. & Zheng, D. Lun qingzanggaoyuan de fanwei he mianji. *Geogr. Res.* 1–8 (2002).

17. Latham, J., Cumani, R., Rosati, I. & Bloise, M. Global land cover share (GLC-SHARE) database beta-release version 1.0-2014. *FAO Rome Italy* **29**, (2014).

18. Jenkerson, C., Maiersperger, T. & Schmidt, G. L. eMODIS: A user-friendly data source: U.S. Geological Survey Open-File Report 2010–1055. (2010).

19. National Bureau of Cultural Relics. *Zhongguo wenwu dituji: Xizang zizhiqu fence*. (Wenwu chubanshe, 2010).

20. National Bureau of Cultural Relics. *Zhongguo wenwu dituji: Qinghai fence*. (Wenwu chubanshe, 1996).

21. National Bureau of Cultural Relics. *Zhongguo wenwu dituji: Yunnan fence*. (Yunnan keji chubanshe, 2001).

22. National Bureau of Cultural Relics. *Zhongguo wenwu dituji: Sichuan fence*. (Wenwu chubanshe, 2009).

23. Jia, X. *Qinghaisheng dongbeibu diqu xinshiqi qingdai shidai wenhua yanhua guocheng yu zhiwuyicun yanjiu*. (Doctoral dissertation, Lanzhou University, 2012).

24. Lu, H. *et al.* Early agropastoral settlement and cultural change in central Tibet in the first millennium BC: excavations at Bangga. *Antiquity* **95**, 955–972 (2021).

25. Borck, L., Mills, B. J., Peeples, M. A. & Clark, J. J. Are social networks survival networks? An example from the late pre-Hispanic US Southwest. *J. Archaeol. Method Theory* **22**, 33–57 (2015).

26. Mills, B. J. *et al.* Transformation of social networks in the late pre-Hispanic US Southwest. *Proc. Natl. Acad. Sci.* **110**, 5785–5790 (2013).

27. Peeples, M. A. Finding a place for networks in archaeology. *J. Archaeol. Res.* **27**, 451–499 (2019).

28. Lu, H. *Kuayue Ximalaya de Wenhua Hudong*. (Kexue Chuban She, 2015).

29. Peeples, M. A. & Roberts Jr, J. M. To binarize or not to binarize: relational data and the construction of archaeological networks. *J. Archaeol. Sci.* **40**, 3001–3010 (2013).

30. Athenstädt, J. C., Mills, B. J. & Brandes, U. Social networks and similarity of site assemblages. *J. Archaeol. Sci.* **92**, 63–72 (2018).

31. de Groot, B. G. A diachronic study of networks of ceramic assemblage similarity in Neolithic Western Anatolia, the Aegean and the Balkans (c. 6600–5500 bc). *Archaeometry* **61**, 600–613 (2019).

32. Coward, F. Small worlds, material culture and ancient Near Eastern social networks. in *Social brain, Distributed Mind* 449–79 (Oxford University Press, 2010).

33. Borgatti, S. P., Everett, M. G. & Freeman, L. C. Ucinet for Windows: Software for social network analysis. *Harv. MA Anal. Technol.* **6**, 12–15 (2002).

34. Freeman, L. C. Centrality in Social Networks Conceptual Clarification. *Soc. Netw.* **1**, 215–239.

35. Csardi, G., Nepusz, T., & others. The igraph software package for complex network research. *InterJournal Complex Syst.* **1695**, 1–9 (2006).

36. Borgatti, S. P., Mehra, A., Brass, D. J. & Labianca, G. Network analysis in the social sciences. *science* **323**, 892–895 (2009).

37. Mizoguchi, K. Nodes and edges: A network approach to hierarchisation and state formation in Japan. *J. Anthropol. Archaeol.* **28**, 14–26 (2009).

38. Lulewicz, J. The social networks and structural variation of Mississippian sociopolitics in the southeastern United States. *Proc. Natl. Acad. Sci.* **116**, 6707–6712 (2019).

39. Roberts Jr, J. M., Yin, Y., Dorshorst, E., Peeples, M. A. & Mills, B. J. Assessing the performance of the bootstrap in simulated assemblage networks. *Soc. Netw.* **65**, 98–109 (2021).

40. Qinghai Instituteof Archaeology, Department of History, Northwestern University & Hualong Bureau of Cultural Relics. Qinghai hualongxian banzhuwa kayuewenhua muzang fajuejianbao. *Kaogu* **8**, 27–44 (1996).

41. Huangyuan Museum, Qinghai Team of Archaeology & Qinghai Academy of Social Science Department of History. Qinghai huangyuan dahuazhongzhuang kayue wenhua mudi fajue jianbao. *Kaogu Yu Wenwu* **5**, 4–25 (1985).

42. Qinghai Institute of Archaeology & Chinese Academy of Social Science. Qinghai dulanxian nuomuhong dalitaliha yizhi diaocha yu shijue. *Kaoguxuebao* **1**, 17–41 (1963).

43. Qinghai Institute of Archaeology & Jilin University Department of Archaeology. Qinghai datong huangjiazhai mudi fajuebaogao. *Kaogu* **3**, 193–206 (1994).

44. Qinghai Institute of Archaeology. Qinghai huangzhong xiaxihe panjialiang kayue wenhua mudi. *Kaoguxuejikan* **8**, 28–80 (1994).

45. Qinghai Institute of Archaeology, Sichuan University Department of Archaeology & Chengdu Institute of Archaeology. Qinghai zhiduoxian pukagongma shiguanmu fajue jianbao. *Zangxuexuekan* **16**, 24–40 (2017).

46. Qinghai Institute of Archaeology. Qinghai hualongxian shangbanzhuwa kayuewenhua mudi dierci fajue. *Kaogu* **1**, 51–64 (1998).

47. Qinghai Institute of Archaeology & Hainan Tibetan Folk Art Center. Qinghai guide shanpingtai kayue wenhua mudi. *Kaoguxuebao* **2**, 255–274 (1987).

48. Qinghai Institute of Archaeology. Qinghai xunhua suhusa mudi. *Kaoguxuebao* **4**, 425–449 (1994).

49. Sichuan University Department of Archaeology, Aba Bureau of Cultural Relics & Jiuzhaigou Bureau of Science. Sichuan abazhou jiuzhaigou ashaonao yizhi de diaocha he shijue. *Kaogu* **10**, 60–68 (2017).

50. Sichuan Institute of Archaeology, Kyushu University, Ganzi Bureau of Culture and Tourism & Luhuo Bureau of Culture and Tourism. Sichuan luhouxian galazong yizhi fajue jianbao. *Sichuan Wenwu* **3**, 15–28 (2012).

51. Sichuan Institute of Archaeology & Ganzi Bureau of Culture. Danbaxian zhonglu haneyi yizhi fajue jianbao. in *Sichuan kaogu baogaoji* 59–77 (Wenwu chubanshe, 1998).

52. Sichuan Committee of Cultural Relics & Ganzi Bureau of Culture and Tourism. Sichuan ganzixian jililong gumuzang. *Kaogu* **1**, 28–36 (1986).

53. Yunnan Museum. Yunan deqinxian nagu shiguanmumu. *Kaogu* **3**, 220–225 (1983).

54. Aba Bureau of Cultural Relics, Chengdu Institute of Archaeology & Maerkang Bureau of Culture and Sports. Maerkang xian shidaqiu yizhi shijuejianbao. *Chengdu Kaogu Faxian* **1**, 468–506 (2015).

55. Yunnan Museum. Yunan deqinxian shidi gumu. *Kaogu* **3**, 275–276 (1983).

56. Chengdu Institute of Archaeology, Aba Bureau of Cultural Relics & Qiang Museum of Maowen. *Maoxian yingpanshan shiguanzang mudi*. (Wenwu chubanshe, 2013).

57. Yunnan Museum. Yunan deqinyongzhi faxian de gumuzang. *Kaogu* **4**, 224–228 (1975).

58. Ganzi Team of Archaeology. Sichuan yajiang batang de shibanmu. *Kaogu* **3**, 213–218 (1981).

59. Chinese Academy of Social Science & Tibet Autonomous Region Cultural Relics Management Committee. Xizang gongga changguogou xinshiqi shidai yizhi. *Kaogu* **4**, 1–19 (1999).

60. Chinese Academy of Science. *Qugong in Lhasa*. (Zhongguo dabaikequanshu chubanshe, 1999).

61. Chinese Academy of Social Science, Tibetan Autonomous Region Cultural Relic and Conservation Institute, Ngari Bureau of Relics & Zanda Bureau of Relics. Xizang ali diqu gurujiamu mudi he quta mudi. *Kaogu* **7**, 29–50 (2015).

62. Sichuan University Center for Tibetan Studies, Sichuan University Department of Archaeology, TAR Bureau of Cultural Relics & Ngari Bureau of Culture, Broadcasting and Television. Xizang Zhadaxian Gebusailu mudi diaochabaogao. *Kaogu* **6**, 39–44 (2001).

63. Sichuan University Center for Tibetan Studies, Sichuan University Department of Archaeology & Bureau of Cultural Relics of Tibet Autonomous Region. *Piyang Dongga yizhi kaogubaogao*. (Sichuan renmin chubanshe, 2008).


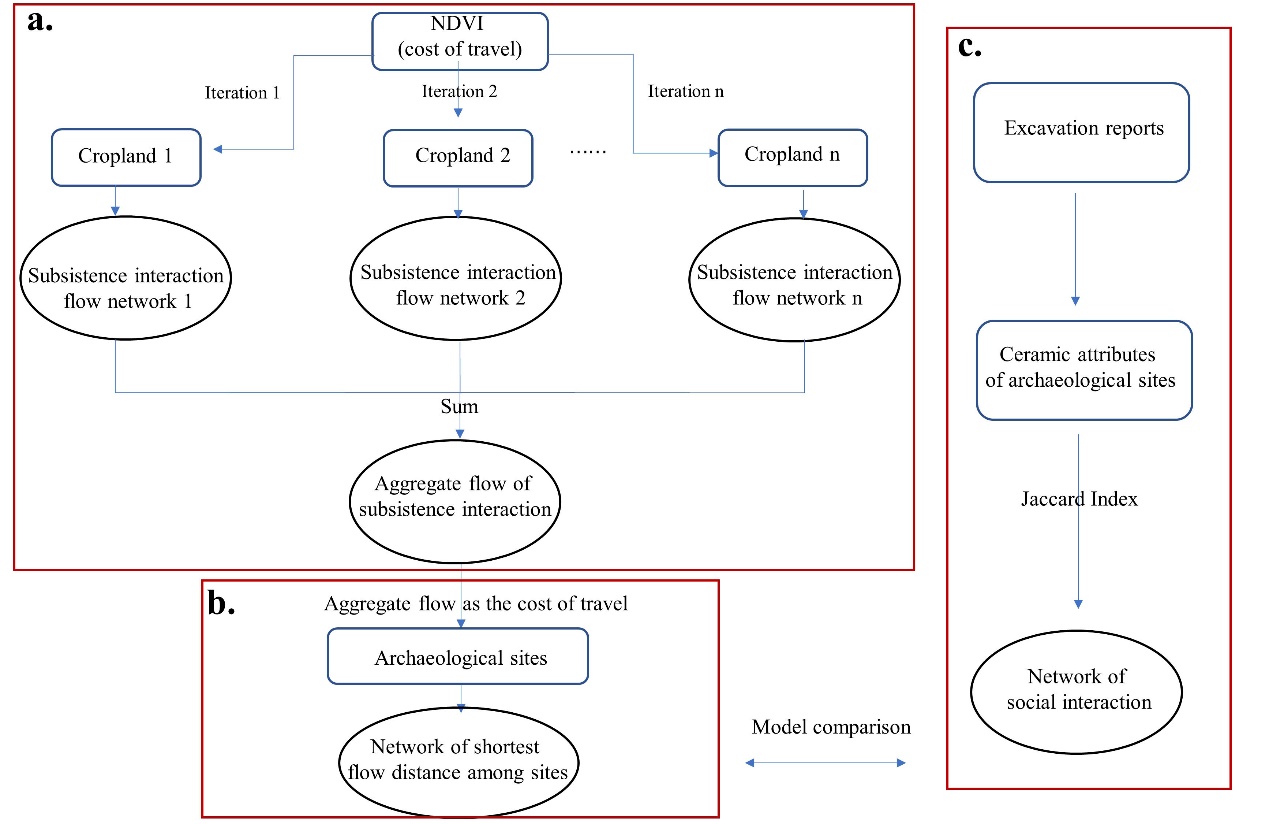


**Supplementary Figure 1.** The diagram of the models and networks in this research. (a) The workflow of the Subsistence Interaction Mobility Model (SIMM). (b) Converting the SIMM into the Subsistence Interaction Network (SIN). (c) The ceramic network to compare with the SIMM and SIN


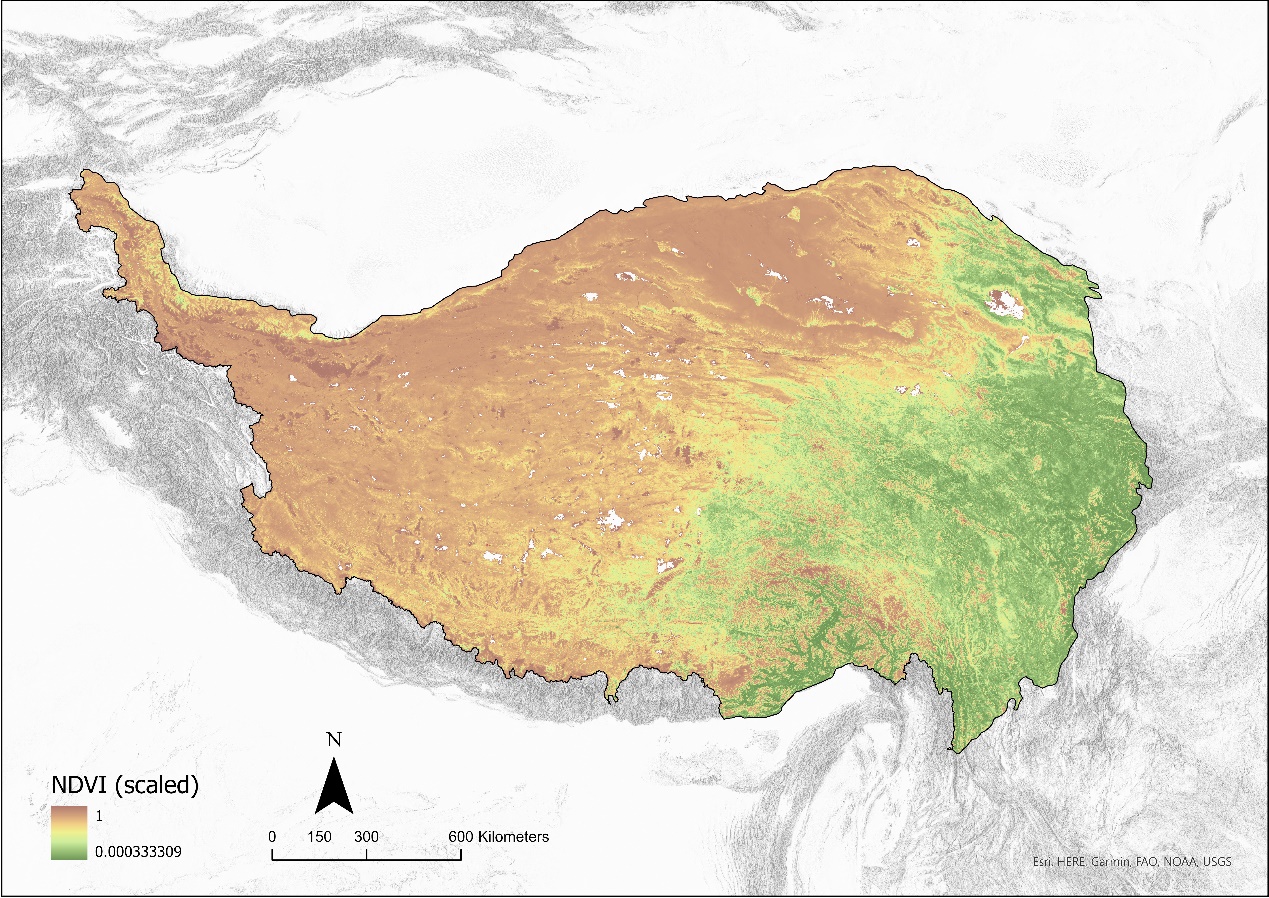
**Supplementary Figure 2.** The scaled NDVI of the Tibetan Plateau used as the cost of travel in the SIMM (Source: USGS EROS Archive eMODIS Remote Sensing Data, 23)


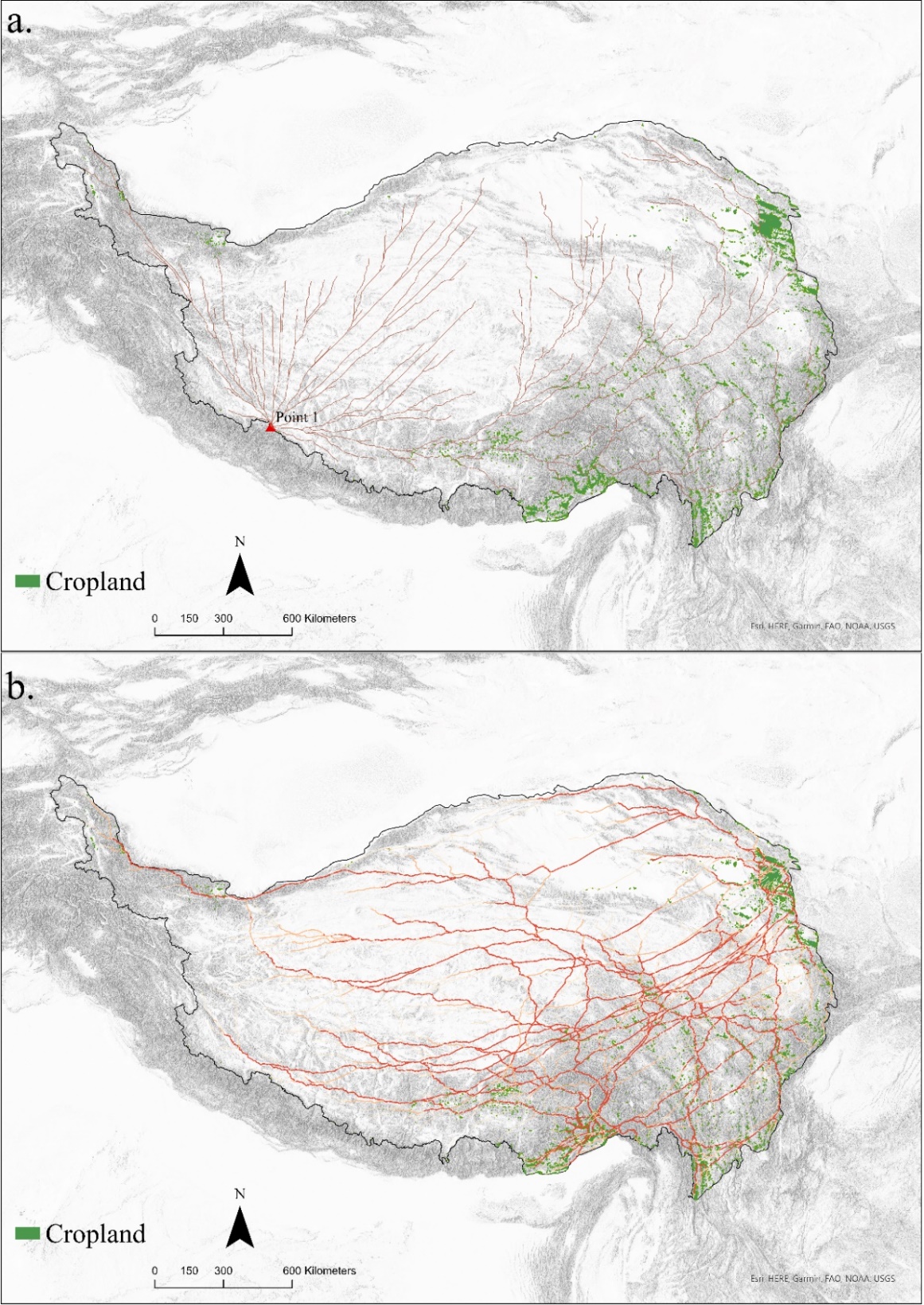


**Supplementary Figure 3.** The workflow of the SIMM. (a) The first iteration of the SIMM simulating the optimal high vegetal pathways towards Cropland Point 1 (the red dot). (b) The SIMM after 6459 iterations showing the aggregate optimal pathways of farming and herding interactions. The red lines are pathways with simulated flow accumulation values greater than one standard deviation (flow value > 540867000, unitless)


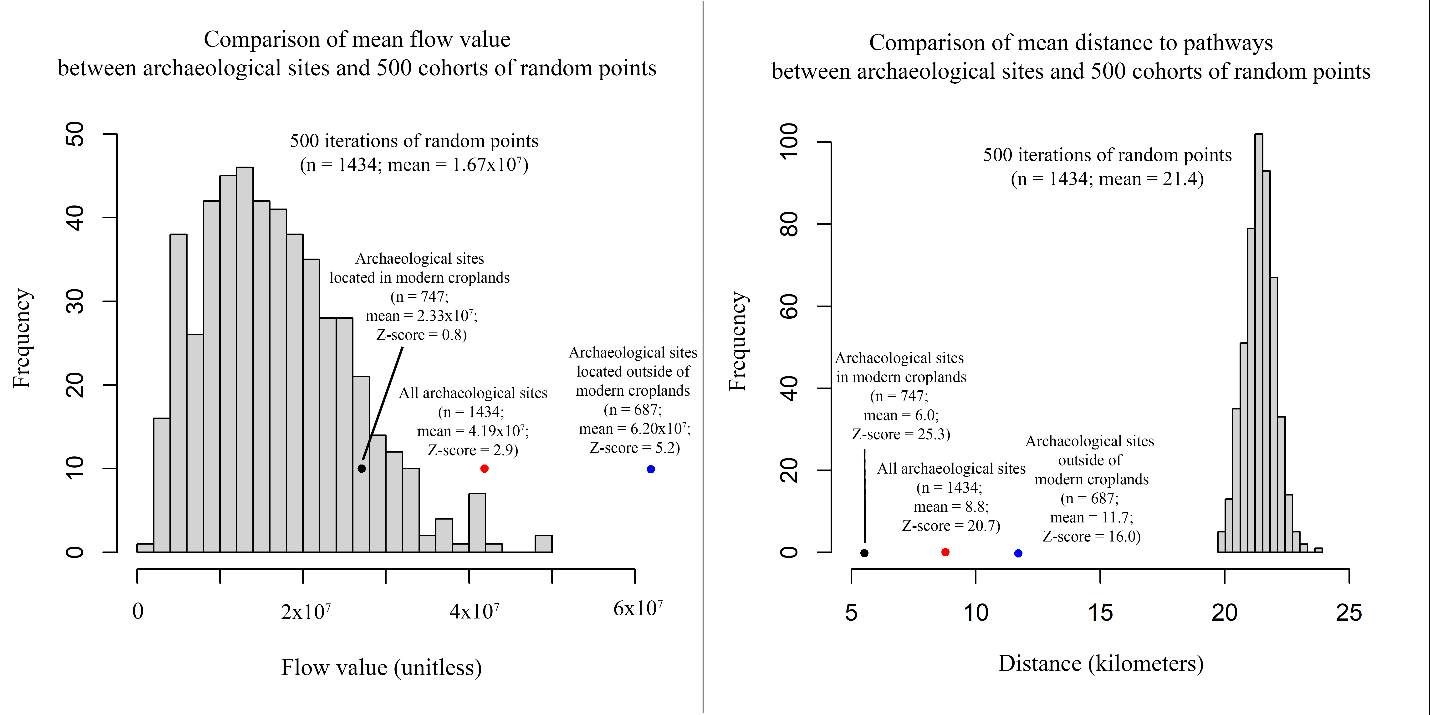


**Supplementary Figure 4**. A heavily annotated version of the statistical validation of the model. All archaeological sites (n=1434) were divided into two groups, archaeological sites within and outside of modern croplands.


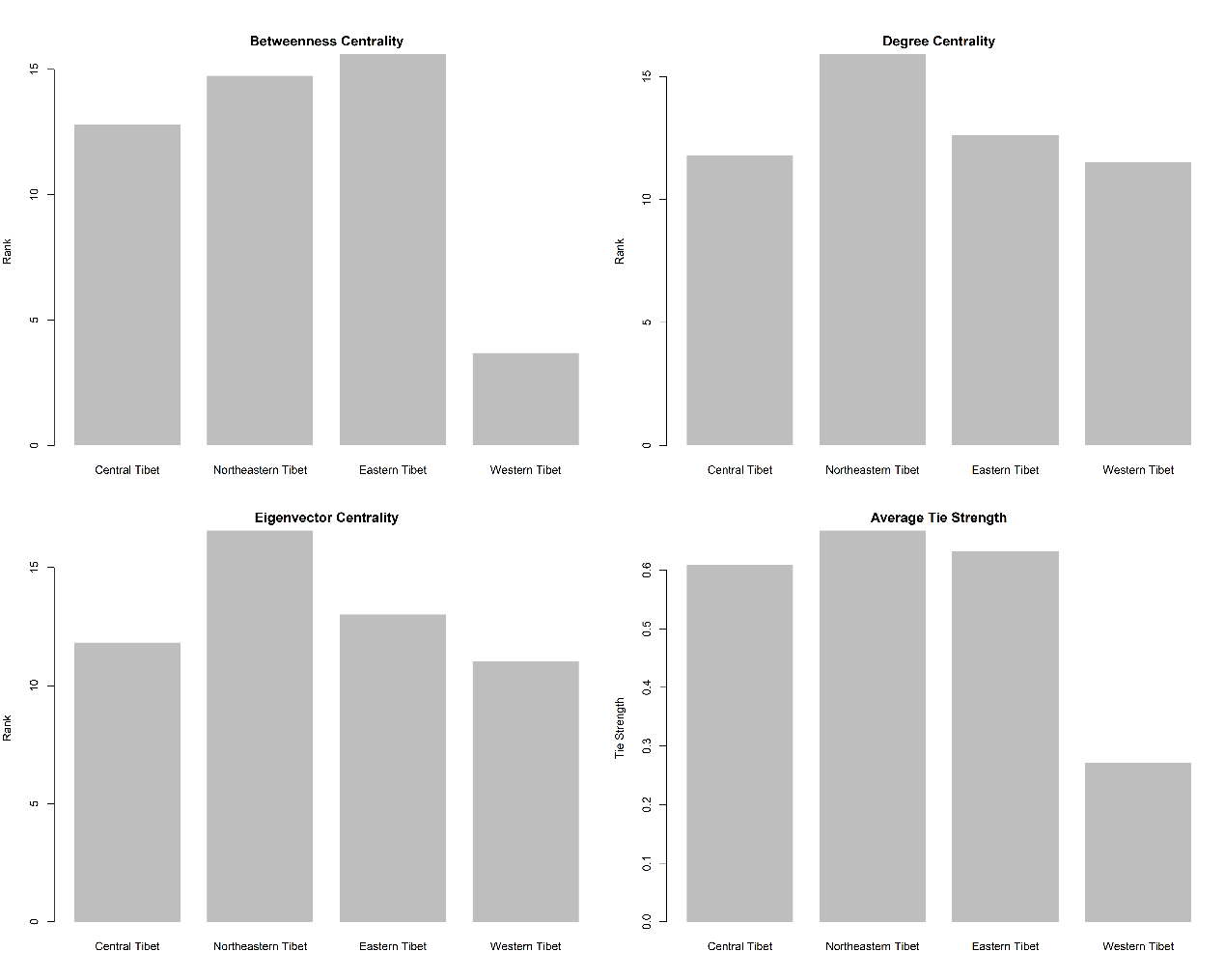


**Supplementary Figure 5**. Comparison of average centrality scores and tie strength among different regions in Tibet in the SIN. The average centrality scores were iteratively calculated under different

threshold values. The results show Western Tibet is a relatively isolated region in the SIN with low centrality scores and weak tie strength.


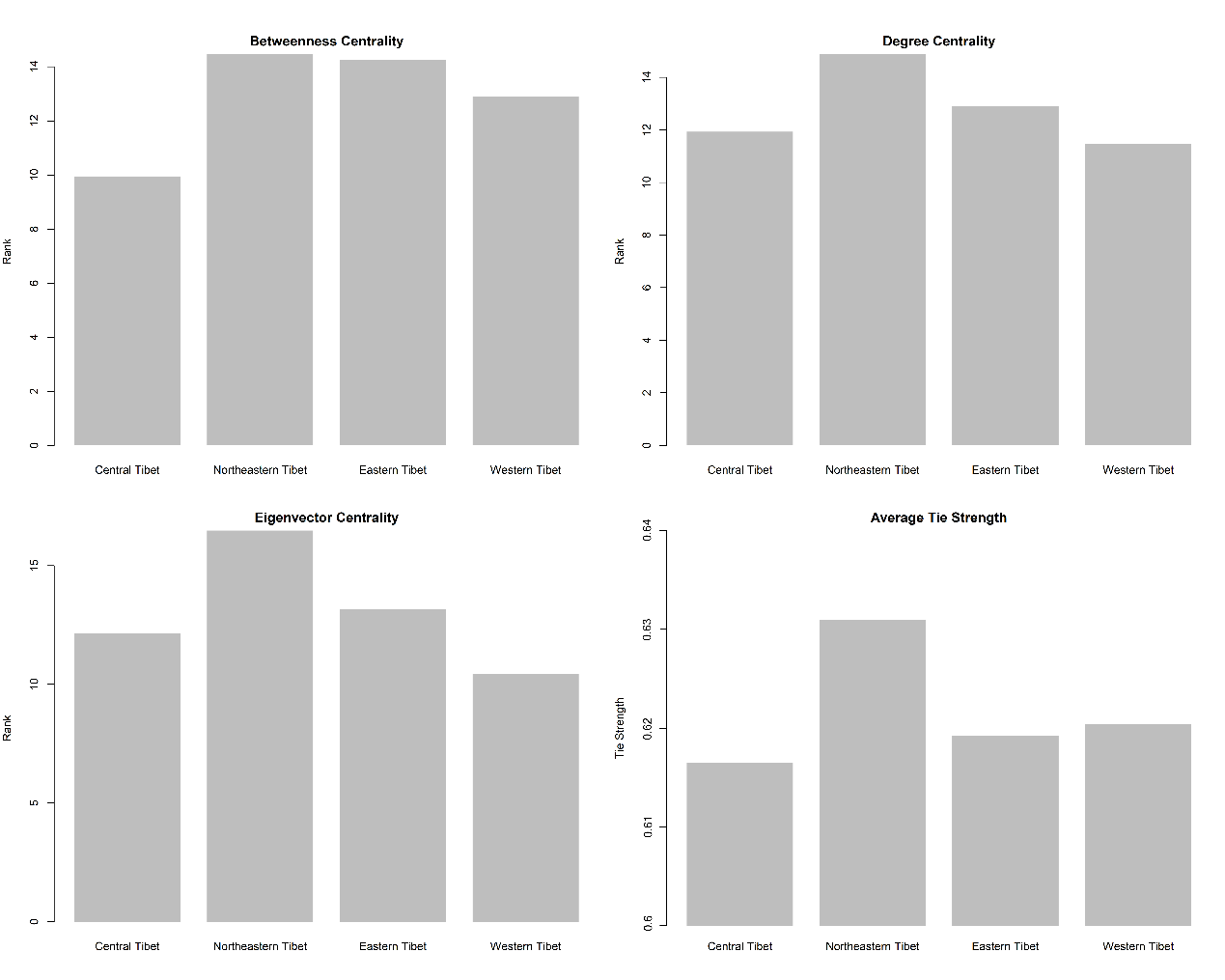


**Supplementary Figure 6**. Comparison of average centrality scores and tie strength among different regions in Tibet in the ceramic network. The average centrality scores were iteratively calculated under different threshold values. The results show that Western Tibet is more tied to the rest of the Tibetan Plateau than modelled in the SIN.

**Supplementary Table 1. The number of ceramics examined in this research**

| **Site** | **Number of ceramics** | **References** |
| --- | --- | --- |
| Banzhuwa | 45 | ^40^ |
| Dahuazhongzhuang | 17 | ^41^ |
| Dalitaliha | 38 | ^42^ |
| Huangjiazhai | 34 | ^43^ |
| Panjialiang | 32 | ^44^ |
| Pukar Gongma | 3 | ^45^ |
| Shangbanzhuwa | 38 | ^46^ |
| Shanpingtai | 34 | ^47^ |
| Suhusa | 23 | ^48^ |
| Ashaonao | 14 | ^49^ |
| Galazong | 10 | ^50^ |
| Haneyi | 10 | ^51^ |
| Jililong | 22 | ^52^ |
| Nagu | 9 | ^53^ |
| Shidaqiu | 98 | ^54^ |
| Shidi | 5 | ^55^ |
| Yingpanshan | 765 | ^56^ |
| Yongzhi | 11 | ^57^ |
| Zhajinding | 23 | ^58^ |
| Bangga | 7963 | ^24^; Artifacts curated at Sichuan University |
| Changguogou | 24 | ^24,59^; Surface collection |
| Jiaritang | 39 | ^59^ |
| Qugong | 227 | ^60^ |
| Chuvthag | 15 | ^61^ |
| Gepaseru | 35 | ^62^ |
| Phiyang_Dungkar | 53 | ^63^ |

**Supplementary Table 2**. **The centrality scores of different sites in the SIN and ceramic network. Arbitrary threshold values were applied to binarize the networks (SIN: 0.68; Ceramic network: 0.63)**

| **Region** | **Site** | **Degree_SIN** | **Eigenvector_SIN** | **Betweenness_SIN** | **Degree_ceramic** | **Eigenvector_ceramic** | **Betweenness_ceramic** |
| --- | --- | --- | --- | --- | --- | --- | --- |
| Northeastern | Banzhuwa | 19.18 | 0.79 | 2.61 | 12.98 | 0.69 | 26.58 |
| Northeastern | Dahuazhongzhuang | 18.99 | 0.78 | 2.61 | 10.79 | 0.59 | 23.08 |
| Northeastern | Dalitaliha | 12.44 | 0.50 | 0.00 | 9.42 | 0.59 | 6.24 |
| Northeastern | Huangjiazhai | 18.91 | 0.78 | 2.61 | 12.26 | 0.67 | 36.79 |
| Northeastern | Panjialiang | 19.03 | 0.79 | 2.61 | 3.96 | 0.23 | 0.00 |
| Northeastern | Pukar Gongma | 24.76 | 0.78 | 127.60 | 3.98 | 0.10 | 4.07 |
| Northeastern | Shangbanzhuwa | 19.19 | 0.79 | 2.61 | 9.98 | 0.59 | 10.45 |
| Northeastern | Shanpingtai | 19.05 | 0.79 | 2.61 | 11.22 | 0.64 | 10.62 |
| Northeastern | Suhusa | 20.09 | 0.82 | 6.39 | 15.09 | 0.87 | 20.58 |
| Eastern | Ashaonao | 24.65 | 0.92 | 30.97 | 0.00 | 0.00 | 0.00 |
| Eastern | Galazong | 18.69 | 0.69 | 11.55 | 6.68 | 0.18 | 7.37 |
| Eastern | Haneyi | 15.90 | 0.58 | 0.00 | 20.64 | 1.00 | 140.43 |
| Eastern | Jililong | 26.87 | 1.00 | 39.24 | 6.89 | 0.35 | 7.77 |
| Eastern | Nagu | 16.79 | 0.57 | 19.30 | 10.86 | 0.42 | 80.43 |
| Eastern | Shidaqiu | 27.03 | 1.00 | 39.24 | 1.31 | 0.11 | 0.00 |
| Eastern | Shidi | 14.97 | 0.54 | 0.00 | 4.06 | 0.08 | 0.67 |
| Eastern | Yingpanshan | 15.10 | 0.56 | 0.00 | 12.49 | 0.68 | 20.02 |
| Eastern | Yongzhi | 15.08 | 0.55 | 0.00 | 4.12 | 0.07 | 1.67 |
| Eastern | Zhajinding | 17.10 | 0.63 | 8.26 | 13.34 | 0.61 | 77.74 |
| Central | Bangga | 8.36 | 0.12 | 11.77 | 3.99 | 0.22 | 4.58 |
| Central | Changguogou | 7.19 | 0.09 | 0.00 | 11.53 | 0.66 | 16.13 |
| Central | Jiaritang | 7.02 | 0.09 | 0.00 | 0.00 | 0.00 | 0.00 |
| Central | Qugong | 7.23 | 0.09 | 0.00 | 11.53 | 0.66 | 16.13 |
| Western | Chuvthag | 3.96 | 0.00 | 0.00 | 6.83 | 0.28 | 11.10 |
| Western | Gepaseru | 3.98 | 0.00 | 0.00 | 5.45 | 0.26 | 2.95 |
| Western | Phiyang_Dungkar | 3.96 | 0.00 | 0.00 | 10.80 | 0.59 | 18.61 |

| **Region** | **Average**  **Degree_SIN** | **Average**  **Eigenvector_SIN** | **Average**  **Betweenness_SIN** | **Average**  **Degree_ceramic** | **Average**  **Eigenvector_ceramic** | **Average**  **Betweenness_ceramic** |
| --- | --- | --- | --- | --- | --- | --- |
| Northeastern | 19.07 | 0.76 | 16.63 | 9.96 | 0.55 | 15.38 |
| Eastern | 19.22 | 0.70 | 14.86 | 8.04 | 0.35 | 33.61 |
| Central | 7.45 | 0.10 | 2.94 | 6.76 | 0.39 | 9.21 |
| Western | 3.97 | 0.00 | 0.00 | 7.69 | 0.37 | 10.89 |

**Supplementary Table 3**. **The average centrality scores of different regions in the SIN and ceramic network. Arbitrary threshold values were used t**

**o binarize the networks (SIN: 0.68; Ceramic network: 0.63)**
